# Empowering genome-wide association studies via a visualizable test based on the regional association score

**DOI:** 10.1073/pnas.2419721122

**Published:** 2025-02-25

**Authors:** Yiran Jiang, Heping Zhang

**Affiliations:** ^a^Department of Biostatistics, Yale University, New Haven, CT 06511

**Keywords:** genome-wide association study, regional association, polygenic risk scores

## Abstract

The genome-wide association studies identified genes associated with many diseases, but the identification and verification of disease variants are still challenging due to small effects and large number of individual variants. In this paper, we propose a powerful method that first quantifies the strength of regional associations at each single nucleotide polymorphism and converts these measures into time series data before using a change point detection algorithm to identify significant regions. In our extensive simulation study, the proposed method consistently demonstrates greater power than existing alternatives, achieving a relative increase of over 20% in challenging scenarios where true causal variants are sparse and multiple association regions exist at the same time, while maintaining a lower false positive rate.

## Significance

Genome-wide association studies (GWAS) are crucial for identifying numerous single nucleotide polymorphisms (SNPs) linked to various diseases. However, current methods struggle with regional associations due to small effects and the high number of variants, leading to suboptimal power and inflated type I error. To tackle these challenges, we propose a powerful and visualizable method which quantifies regional association strengths at individual SNPs, converts these into time series data, and uses change point detection algorithms to identify key association regions. Extensive simulations demonstrate that our method not only increases detection power but also maintains a significantly lower false positive rate compared to existing techniques, positioning it as a promising tool for regional association detection in GWAS.

Genome-wide association studies (GWAS) have successfully identified numerous single nucleotide polymorphisms (SNPs) associated with many diseases. Traditionally, these associations are tested by examining each SNP individually in relation to the phenotype of interest, necessitating stringent multiple-testing thresholds to mitigate false positives (1, 2). However, each SNP generally contributes a small effect which may be difficult to detect even for a large study of hundred of thousand participants (3). It has been observed that many common variants with small effects might cumulatively explain a substantial proportion of the heritability (4–6). Reflecting this understanding, recent research has shifted focus from analyzing individual SNPs to examining groups of SNPs within specific regions (7–9).

The most common method for detecting potential association regions involves postinspection, which typically includes visualizing the GWAS statistics using software such as LocusZoom to capture regional information (10–12). Although this approach is straightforward and effective, it is subjective and may sometimes either misattribute significance to some loci or neglect others that are significant (8). Alternatively, set-based testing methods, adapted for GWAS, are another approach for detecting regional associations (13, 14). This category includes methods such as the burden tests (2, 15), sequence kernel association test (SKAT) (16), SKAT-O (17, 18), RC-SKAT (4), and others (13, 19, 20). The advantage of these methods is that they can effectively aggregate the small effects of causal variants in a region, which can increase the power. However, despite being highly versatile, these group-based testing methods often require prior information of the effect sizes of causal variants, which can influence the power of the tests (16) and the results are not easy to interpret due to their aggregate nature.

In this paper, we propose a powerful and user-friendly regional association detection method that combines the visualization strength of the postinspection method and the analytical rigor of set-based testing approach. This method not only automates the detection of potential association regions but also facilitates the visualization of association strength around each SNP position on the chromosome through trend plots suitable for postinspection. More importantly, unlike the existing set-based testing methods, our method overcomes the inflated type I error when there exist causal variants and does not require prior knowledge of the effect sizes of causal variants. The key idea of our method is to leverage the localized polygenic risk score (LPRS), which becomes the polygenic risk score (PRS) (21–23) if it is not localized, and we adapt the LPRS to aggregate the effect of the SNPs within a scanning window with an adaptive window size centered on each pivotal SNP. The strongest association, measured between the aggregated PRS and the phenotype within this adaptive window, is termed the regional association score (RAS). This score represents the regional association strength of each pivotal SNP.

By selecting the pivotal SNPs at regular intervals along a chromosome, we transform the RASs of these pivotal SNPs into times series data and then apply the change point detection algorithm for automatically detecting these potential association regions. Extensive simulation studies demonstrate that our proposed method provides greater power than the existing set-based testing methods adapted for GWAS. Moreover, it can much more effectively control the false positive rate (detect incorrect regions) when there exist true signals across the chromosome. Furthermore, an application of our method for the Adolescent Brain Cognitive Development^SM^ (ABCD) study® (24, 25) has successfully identified association regions with support in the literature.

## Results

### Overview of the Proposed Method Based on RAS.

**Fig. 1.**
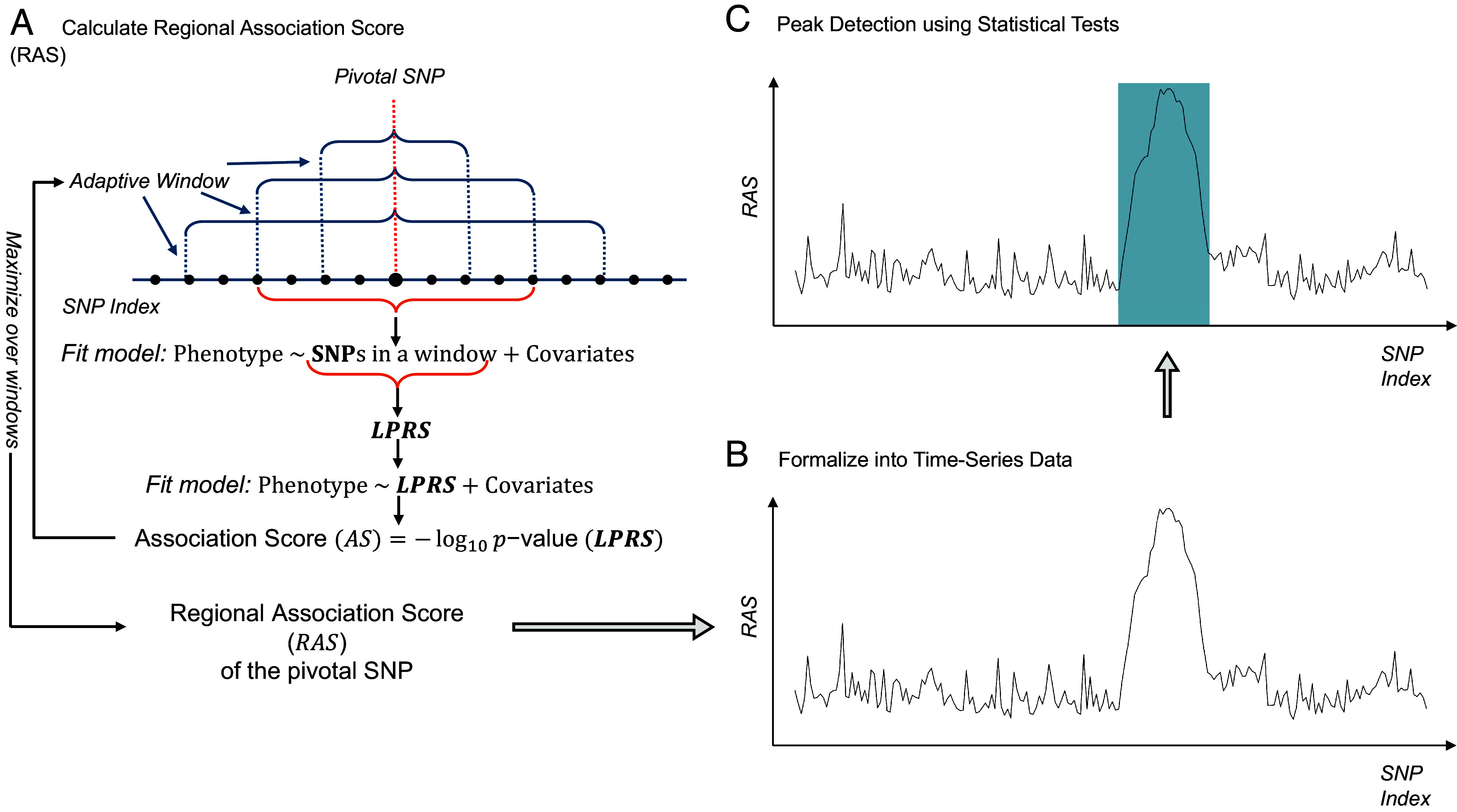
Graphical overview of the RAS method. (*A*) An adaptive window aggregates the genetic influence around a pivotal SNP. The window size is optimized to achieve the highest Association Score (AS) through regression analysis with the phenotype. This score is then designated as the RAS for the pivotal SNP. (*B*) By systematically selecting the pivotal SNP across the entire chromosome, the RAS and the positions of these SNPs are formulated into time series data. (*C*) Finally, peak detection methods utilizing statistical tests are applied to identify regions of significant association, which are visualized as peaks in the data.

Fig. 1 provides a graphical overview of our regional association detection method utilizing the RAS. An adaptive window captures the aggregated genetic effects within a region surrounding a pivotal SNP, and the association strength between these effects and the target phenotype is quantified as the RAS for that SNP. By selecting pivotal SNPs to span the entire chromosome, the calculated RAS values, along with their SNP positions, are formulated into time series data. The regions that have underlying association with the target SNP are shown as peaks. Subsequently, peak detection methods based on statistical tests are then utilized to identify such peaks and mark them as associated regions. Detailed descriptions of the problem formulation and the implementation of the method are provided in the *Materials and Methods* section of the paper.

In line with GWAS and traditional PRS methodologies, our approach primarily targets the association study of common and low-frequency variants, specifically those with a minor allele frequency (MAF) of 1% or higher, as typically observed in genotyping data. Recent advancements in PRS methodologies suggest a growing interest in incorporating rare variants (26) (MAF < 1%), which may represent a promising direction for future extensions of our methods. For the scope of the present study, we concentrate on analyzing SNPs with MAF ≥ 1%.

### The RAS Method Overcomes the Inflated Type I Error and Performs Robustly with Limited Prior Information on SNP Effects.

For the set-based testing methods that are used to detect the significant association regions, there are two main notable limitations: First, the presence of causal variants can increase the probability that nonassociated regions on the same chromosome being mistakenly classified as significantly associated—a phenomenon known as inflated type I error—due to elevated test statistics resulting from linkage disequilibrium (LD) (27, 28). Second, these methods generally require prior information on SNP effects, which significantly influences their power. For example, in SKAT, parameters are prespecified to enhance power under the assumption that the rarer variants are expected to be more likely to have larger effects.

In contrast, the proposed RAS method identifies significant regions by comparing the relatively high RAS values of these regions to others, making them stand out as peaks. Theoretical discussion about such an observation is provided in *SI Appendix*, Section S.1.1. This approach mitigates the impact of inflated test statistics caused by LD, as it focuses on relative rather than absolute values. Our simulation studies, detailed in the subsequent section, demonstrate that the RAS method significantly reduces type I error compared to set-based methods in scenarios involving causal variants on the chromosome. Theoretical intuitions are provided in *SI Appendix*, Section S.1.2. Additionally, the RAS calculation does not depend on assumptions beyond the basic linear effects model, thus decoupling the method's power from prior information and enhancing its robustness.

### Simulation Study.

We conducted simulation studies in various settings to evaluate the ability of our proposed method to control type I error and compare its power with existing methods. These simulations utilized genotyping data from release 5.0 of the Adolescent Brain Cognitive Development^SM^ (ABCD) study®. The ABCD study genotyping array data have been playing a crucial role in various genetic association studies since its first release (29, 30). The Affymetrix Axiom Smokescreen Array, which serves as the genotyping platform, is specifically designed to cover the whole genome while focusing on variants related to substance use and diverse global ancestry (31). A detailed introduction to the genotyping protocol is provided by Fan et al. (30). In our simulation study, we used data from chromosome 21, which includes 5,718 SNPs for a cohort of 11,650 individuals, to generate the underlying true phenotype data. The SNP data do not contain rare variants, and the percentage of low-frequency (1% ≤ MAF < 5%) and common variants (MAF ≥ 5%) are 20% and 80%, respectively. An illustrative distributional histogram is presented in *SI Appendix*, Fig. S5. To simplify the analysis, common confounding covariates such as age and sex were assumed to be independent of the phenotype data, obviating the need for adjustments for these confounders in our simulation study.

### Type I Error Rate.

We considered both continuous and dichotomous phenotypes. Continuous phenotypes were generated from the null linear model:

$$Y=\alpha_{0}+\epsilon ,\;\epsilon ∼N\left(0,\sigma^{2}\right),$$

with σ=1 and the intercept term $\alpha_{0}=0$. Dichotomous phenotypes were generated from the null logistic model:

$$\mathrm{logit}\;P\left(Y=1\right)=\alpha_{0},$$

where $\alpha_{0}$ is the intercept term used to control the simulated proportion of approximately 0.2 cases and 0.8 controls. It is a common practice to use the intercept term to manage the proportion of cases to controls, where cases are typically less than or equal to 0.5 (15–18). The proposed algorithm was applied to the genotype data from chromosome 21 of the ABCD study, with phenotypes simulated according to these null models. A false positive is recorded when the detection algorithm incorrectly identifies any region of the chromosome as being significantly associated with the phenotype at the 0.05 level over the entire chromosome. Given that this target identification level applies to the entire chromosome rather than a single SNP, no additional Bonferroni adjustments are necessary.

### Power.

The assessment of power is conducted on the same chromosome, using the models based on alternative hypotheses, which posit an association between certain SNPs on the chromosome and the phenotype. Specifically, with the SNP matrix G and the coefficient β defined previously, for the alternative model with continuous phenotype, we use

$$Y_{i}=\alpha_{0}+\beta \prime G_{i}+\epsilon_{i},\;\epsilon_{i}∼N\left(0,\sigma^{2}\right),$$

with σ=1 and the intercept term $\alpha_{0}=0$. The alternative model with dichotomous phenotype is defined as

$$\text{logit}\;P\left(Y_{i}=1\right)=\alpha_{0}+\beta \prime G_{i},$$

where $\alpha_{0}$ sets the n simulated phenotype values with a proportion of approximately 0.2 cases and 0.8 controls.

We specify that the true coefficient β reflects localized patterns in $M\;\left(\text{where}\;M>0\right)$ regions, each containing T (where T>0) consecutive SNPs. Specifically, we let the true causal variants be located in these M regions, with the effect size correlated to the MAF of the SNP. Following the setting in Wu et al. (16), we set the magnitude of each $\beta_{j}$ to $c|{\text{log}}_{10}\;MAF_{j}|$ with some selected signal strength parameter c, assuming that the effects of causal variants decrease with MAFs. Moreover, each $\beta_{j}$ takes positve or negative sign randomly. We further define $q\in \left(0,1\right)$ to represent the proportion of causal variants in these regions. Specifically, with the number of signals M, signal window size T and causal variants rate q, the coefficients are determined as

$$\begin{array}{c} \beta_{j}=\left\{\begin{array}{c} \pm s\cdot c\cdot |{\text{log}}_{10}\;MAF_{j}|\;\text{for}\;j\in \left\{s_{m},\cdots ,s_{m}+T-1\right\}\;\text{and}\;m\in \left\{1,\cdots ,M\right\}\\ \;0\;\text{Otherwise} \end{array}\right., \end{array}$$

for j=1,⋯,p. Here, $s_{m}$ indicates the starting index of the signal region $m\in \left\{1,\cdots ,M\right\}$ and s is a Bernoulli random variable with parameter q. In our simulations, we set the signal window size T=100, resulting in an average association region length of approximately 0.3 Mb. This length aligns numerically with findings from existing literature, where reported association region lengths typically range from about 0.1 Mb to 1 Mb (3, 32). For the signal strength, we set c=0.04 for the continuous phenotype and c=0.08 for the dichotomous phenotype. We vary $M\in \left\{1,3\right\}$ and $q\in \left\{1,0.5,0.3\right\}$ for different simulation settings.

In our evaluation of power, we assess the ability of our proposed method to accurately identify all M association regions on the chromosome. The significant level threshold applied here is based on the null model, which maintains the type I error rate to be lower than (and close to) 0.05. An association region is deemed correctly identified if it overlaps with any of the regions detected by our method. Additionally, we assess the false positive rate under the alternative model (FPR-A). A false positive is recorded when a detected region does not overlap with any true association regions.

### Existing Methods.

Several effective set-based association testing methods, burden tests (2, 15), SKAT (16), SKAT-O (17, 18), RC-SKAT (4), CauchyGM, and CauchyGM-O (33) are included for comparison. While burden test, SKAT, and SKAT-O are primarily designed for analyzing low-frequency and rare variants, they are also applicable to common variants, offering an alternative to the traditional GWAS (34). RC-SKAT, CauchyGM, and CauchyGM-O are successors that have proven effective in handling common and low-frequency (1% or higher) variants. These methods were applied to detect potential association regions using the moving-window approach suggested by Lee et al. (17) and Price et al. (35). Specifically, the statistical association tests are conducted within a moving-window that scans the whole chromosome. For each window, a *P*-value is calculated to represent the association significance of the region it spans. A region is deemed significantly associated with the phenotype if its *P*-value falls below a threshold value, denoted by α, which is set to control the type I error. If a true signal region overlaps with these detected significant regions, the detection is considered valid, meaning no false negative occurs at this region. A detected significant region that does not overlap with any true signal regions is considered a false positive. In favor of these methods, the moving-window size is set at 100, which matches T, the size of the true signal window. The *P*-value threshold α is selected to control the type I error to be lower than (and close to) 0.05.

### Simulation Results.

**Fig. 2.**
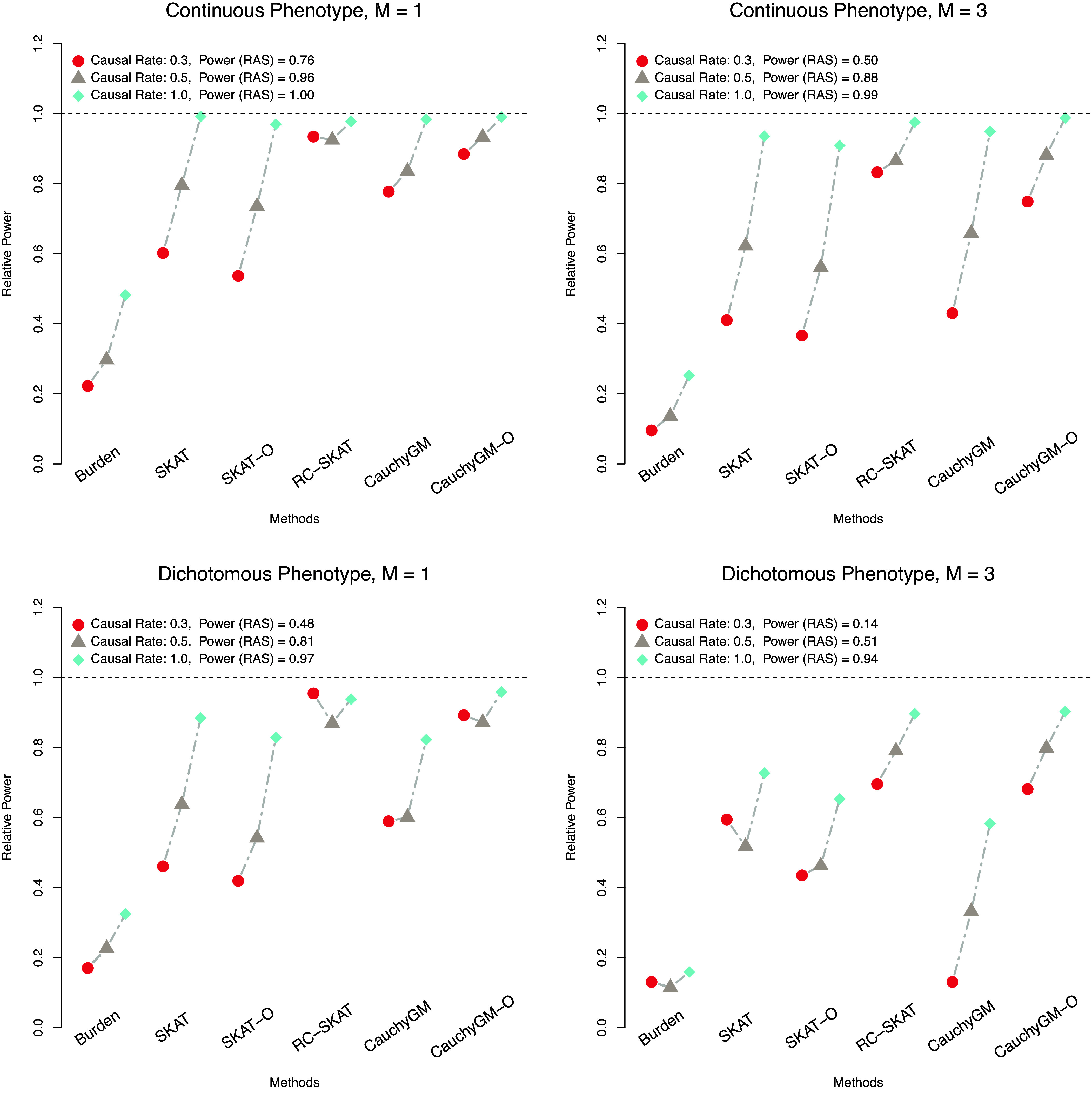
Comparison of the power estimates for the five methods involved in the simulation study. The power of our proposed RAS method indicated above the horizontal dotted lines is used as the reference, and the *y* axis is the ratio of the power of each alternative method to that of RAS.

**Fig. 3.**
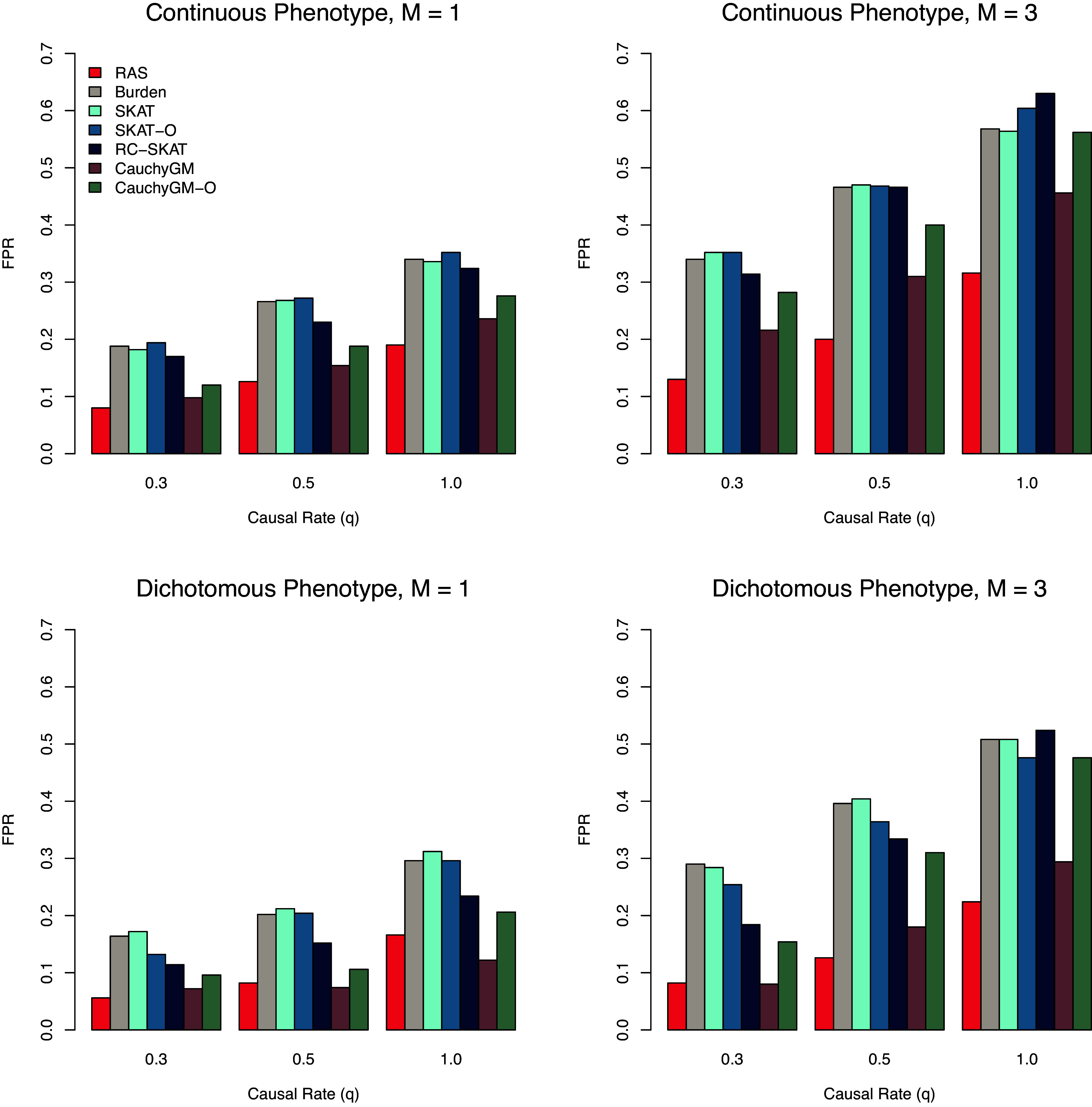
Comparison of the FPR-A for the five methods involved in the simulation study.

The type I error results for all methods based on 500 repetitions under the null models are summarized in Table 1, which shows that all methods have effectively controlled the type I error to be less than 0.05 over the entire chromosome with their selected threshold values. For all methods, the same *P*-value threshold that yielded Table 1 is used to evaluate the power and the FPR-A. The comparison of power and FPR-A between different methods is demonstrated in Figs. 2 and 3, respectively. It is shown that the proposed method numerically outperforms all other methods in all cases. Specifically, our proposed method identified more true association regions than the competing methods, while maintaining a much lower FPR-A. Notably, the superiority of our proposed method becomes more evident in challenging settings where the ratio of causal variants is low and multiple association regions are present on the chromosome. In these scenarios, the best alternative methods achieve only 80% (for continuous traits) and 70% (for dichotomous traits) of the power of our method, while almost doubling the FPR-A. These results highlight the effectiveness of our proposed method in accurately identifying regional genetic associations.

**Table 1.** Summarized type I error rates on the null model with different methods, estimated with 500 runs

	RAS	Burden	SKAT	SKAT-O	RC-SKAT	CauchyGM	CauchyGM-O
Continuous	0.042 (0.009)	0.046 (0.009)	0.050 (0.010)	0.050 (0.010)	0.050 (0.010)	0.050 (0.010)	0.048 (0.010)
Dichotomous	0.044 (0.009)	0.050 (0.010)	0.050 (0.010)	0.048 (0.010)	0.050 (0.010)	0.050 (0.010)	0.050 (0.010)

The SD are given in parentheses. The experiment is conducted on chromosome 21 of the ABCD data, containing 5,718 SNPs.

As a note on computational efficiency using a single average CPU, our proposed method requires approximately 2 min for each repetition in the continuous case and 6 min in the dichotomous case. In comparison, the existing set-based testing methods take about 3 min for continuous cases and 4 min for dichotomous cases. While our method demonstrates comparable efficiency to existing approaches, computational time remains to be a practical consideration, particularly with large datasets. There is typically a trade-off between computational accuracy and burden across these methods. We discuss this further along with potential strategies to mitigate computational challenges in *SI Appendix*, Section S.5.

### Application with Real Data.

As an illustrative example, we applied our proposed method to study the genetic associations in the Brief Problem Monitor (BPM) outcomes from the ABCD study, using a cohort of 11,488 individuals, of which 47.7% are female. The cohort’s race/ethnic composition is 52.1% White, 14.8% Black, 20.1% Hispanic, 2.1% Asian, and 10.5% Other. The age distribution is presented in *SI Appendix*, Fig. S7. The BPM is a rating instrument for monitoring children’s functioning and responses to interventions (36), particularly in relation to mental health and depression. For our association studies, we used T-scores obtained from the answers to 20 relevant questions. We include covariates such as age, sex, and the top 10 principle components from the genotype data, processed with the software PLINK (37).

**Fig. 4.**
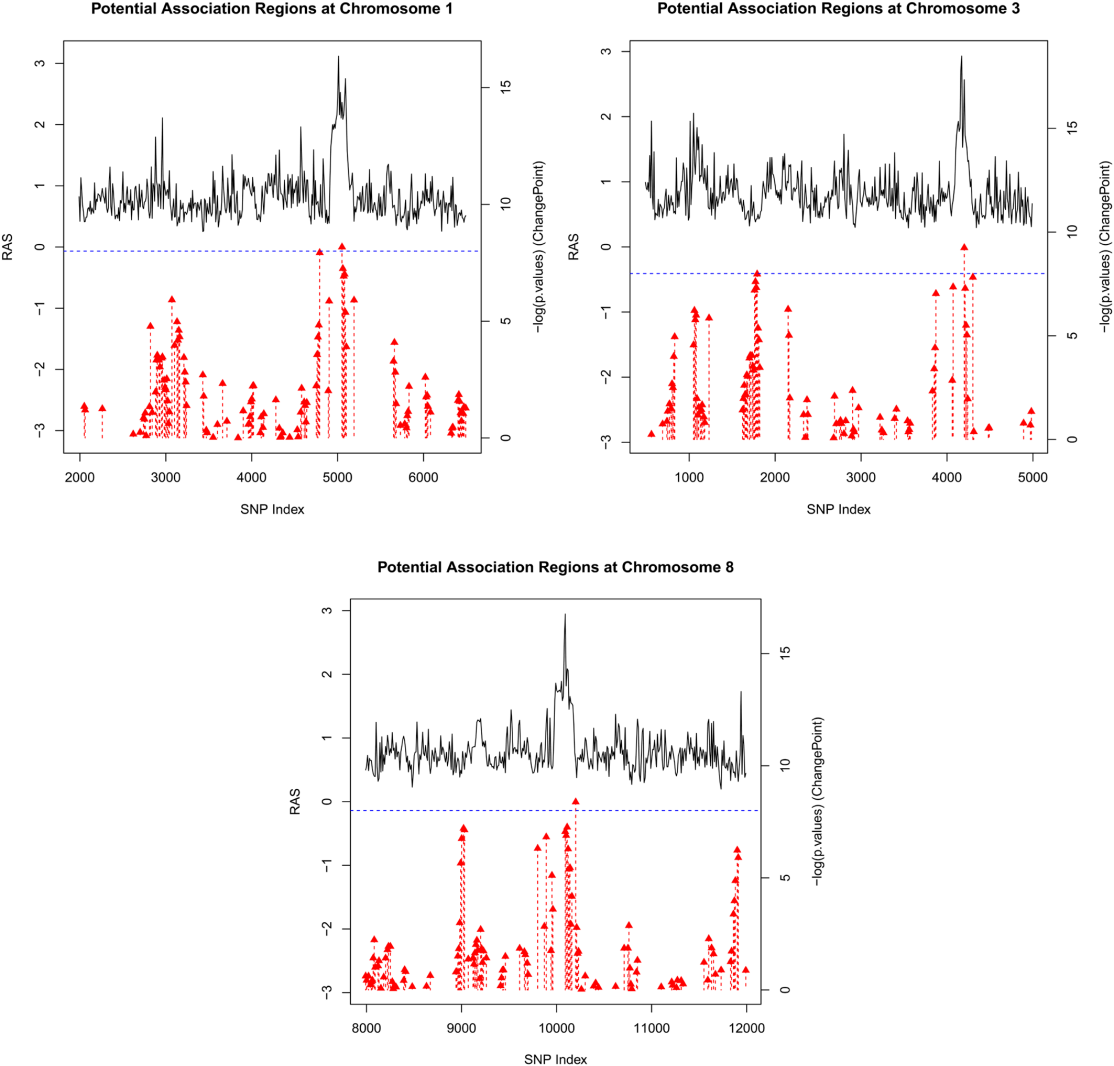
Detected potential association regions on chromosomes 1, 3, and 8. Red dotted lines indicate change points identified in certain sliding windows. Red symbols represent the *P*-value of the test for the existence of a change point within the corresponding sliding window. The blue dotted horizontal line signifies the threshold value of the test. The change points that fail to meet the slope condition are not shown in the plot.

Our method was applied across the entire genome to identify potential association regions. The genotyping data contain 360,717 variants, and the percentages of low-frequency (1% ≤ MAF < 5%) and common variants (MAF ≥ 5%) are 20% and 80%, respectively. An illustrative distributional histogram is presented in *SI Appendix*, Fig. S6. The total running time without parallelization is approximately 2.58 CPU hours. The detected regions on chromosomes 1, 3, and 8 are visualized in Fig. 4. Notably, the detected regions belong to gene *ZNF362*, *SLC18A1*, and *LINC02022* correspondingly. Previous research has identified an association between the gene *ZNF362* and the positive and negative dimensions of the Marder factor scores (38), which are linked to specific psychopathological features such as uncontrolled hostility/excitement and anxiety/depression. Additionally, other studies have shown that genes *SLC18A1* and *LINC02022* are related to lipid measurement (triglycerides and cholesterol) (39, 40), which are further recognized for their connections to depression, anxiety, and stress-related disorders by recent studies (41). The information of the previous genetic association studies for the three identified genes was sourced from the GWAS knowledgebase NHGRI-EBI GWAS Catalog (www.ebi.ac.uk/gwas) (42). This existing evidence reinforce the validity of the potential association regions identified by our method, demonstrating its utility and relevance in uncovering genetic factors related to complex traits.

In addition to the application of our method, benchmark methods were also employed on the same dataset for comparison. Both RC-SKAT and CauchyGM-O identified the gene *ZNF362*, which aligns with the regions detected by our RAS method, thus supporting its validity. However, the genes *SLC18A1* and *LINC02022*, though associated with lipid measurements and potentially linked to psychological traits as mentioned earlier, were not identified by these benchmark methods. Interestingly, SKAT, SKAT-O, CauchyGM, and CauchyGM-O identified the gene *UTC* on chromosome 6. Although there is limited literature supporting its direct association with children’s mental health, this finding suggests additional areas for future investigation. Overall, our method has demonstrated an ability to identify more relevant genetic regions compared to the benchmark methods used in this study. An additional example applying our method to a binary trait using the larger UK Biobank dataset (43) is presented in *SI Appendix*, Section S.7.

## Discussion

In this paper, we proposed a method for detecting potential association regions in GWAS. Unlike traditional single SNP testing, our method aggregates small effects across SNPs within a region, thereby enhancing statistical power. It combines the advantage of the subjective postinspection method and the analytically rigorous set-based testing method. Simulation study has shown that our method not only improves power but also maintains effective control of the FPR compared with an existing test-based method adapted for GWAS. In a practical application using phenotype data from the ABCD study, our approach identified genes potentially associated with specific traits, with these findings confirmed by previous research.

Currently, GWAS primarily relies on single SNP analyses, underscoring the need for robust region-based methodologies. Given the limitations of single SNP testing in capturing the complex genetic architecture of many traits and diseases, further exploration into region-based approaches is well justified. While our method in its current form appears to be promising, due to its expository nature, further empirical and theoretical exploration is necessary. For instance, theoretical considerations can be elaborated further. Moreover, a more effective peak point detection method may be developed to further enhance the method’s power and applicability.

Furthermore, there are several possible extensions of the proposed RAS method. For instance, our method could be adapted for use in transcriptome-wide association studies by leveraging expression quantitative trait loci (eQTLs) data (44, 45). Additionally, the techniques used to calculate the LPRS (23, 46) could be further refined to enhance methodological accuracy and efficiency. A detailed discussion of these potential refinements and extensions is provided in *SI Appendix*, Section S.6.

## Materials and Methods

### Notations and Models.

We consider p SNPs on a chromosome and denote the position of SNP j to be $l_{j}\left(j=1,\cdots ,p\right)$. Assume the SNPs are sorted based on their location on the chromosome such that $l_{1}<l_{2}<\cdots <l_{p}$. Let $G_{n\times p}={\left(g_{1},\cdots ,g_{p}\right)}^{\prime },$ be the genotype matrix of the p SNPs from n subjects and each element takes a value of either 0,1, or 2, depending on the number of copies of the minor allele of a SNP. Let $y_{n\times 1}={\left(y_{1},\cdots ,y_{n}\right)}^{\prime }$ denote the phenotype vector. Furthermore, let $X_{n\times k}={\left(x_{1},\cdots ,x_{k}\right)}^{\prime }$ denote the covariate matrix containing k covariates such as age, sex, and top principal components from the genotype data. Denote the coefficient vector for the SNPs by $\beta_{p\times 1}={\left(\beta_{1},\cdots ,\beta_{p}\right)}^{\prime }$ and the coefficient vector for the explanatory variables by $\alpha_{k\times 1}={\left(\alpha_{1},\cdots ,\alpha_{k}\right)}^{\prime }$. We consider the following linear model (16) as

[1] $$y_{i}=\alpha_{0}+\beta \prime G_{i}+{\alpha \prime X}_{i}+\epsilon_{i},\;\epsilon_{i}∼N\left(0,\sigma^{2}\right),$$

when the phenotypes are continuous, and logistic model (16) as


[2] $$\text{logit}\;P\left(y_{i}=1\right)=\alpha_{0}+\beta \prime G_{i}+\alpha \prime X_{i},$$

when the phenotypes are dichotomous, where **G_i_** denotes the i-th row of G.

### LPRS and RAS.

Here, we propose an approach to measuring the regional association utilizing LPRS. Specifically, for a small local region on a chromosome containing a set of K consecutive SNPs, indexed by $I_{1},\cdots ,I_{K}\in \left\{1,\cdots ,p\right\},$ the LPRS for the i-th subject is calculated as

[3] $$LPRS_{i}=\sum_{j=1}^{K}{\hat{\beta }}_{I_{j}}G_{iI_{j}},$$

where $G_{iI_{j}}$ represents the $I_{j}$-th entry of **G_i_** and ${\hat{\beta }}_{I_{j}}$ is the estimated effect size of the $I_{j}$-th SNP. Once the LPRS is computed, it can be used to evaluate the association strength between the LPRS and specific phenotypes to assess the impact of genetic liability within the small region on their relationship. This process, defined by the function $f\left(∙,∙\right)$, yields the association measure:

[4] $$AS=f\left(LPRS,y\right),$$

where $LPRS=\left(LPRS_{1},\cdots ,LPRS_{n}\right)\prime$ denotes the vector of LPRS calculated from [**3**]. The evaluation of this association strength can be approached in many ways. In this study, we use a simple approach by fitting a linear regression or logistic regression model y∼LPRS+X, which takes general form as model [**1**] or [**2**] depending on the phenotype data type being continuous or dichotomous. The significance of the LPRS in the model is tested using a t-test, from which the P-value, denoted by P, is obtained. We then use the transformed value $-\log_{10}P$ as the association strength measure, AS.

Unlike traditional methods that assess sets of SNPs, our method evaluates regional association at the level of individual SNPs. Utilizing the LPRS introduced above, our proposed method is implemented in the following steps: First, we construct an adaptive window centered at a single SNP. The effects of the subset of SNPs within this window are aggregated by calculating the LPRS using this subset of SNPs with [**3**]. Meanwhile, this LPRS is used to calculate the association strength with the phenotype utilizing [**4**]. Finally, the optimal window size, which produces the strongest association as measured by the method in Eq. **4** is selected. This last step is not only critical but also distinguishes our method from the others. This distinction is the reason why our method has superior power. The resulting association score represents the RAS for that specific SNP. Formally, for an SNP indexed as j, its RAS is defined as

$$RAS_{j}=\underset{\;t\in T}{\text{max}}f\left(\sum_{k=\text{max}\left(j-t,\;1\right)}^{\text{min}\left(j+t,\;p\right)}{\hat{\beta }}_{k}g_{k},y\right),$$

where $g_{k}={\left(G_{1k},\cdots ,G_{nk}\right)}^{\prime }$ denotes the k-th column of the SNP matrix G, and $f\left(∙,∙\right)$ is the function that measures the association strength between the LPRS and the phenotype, elaborated following Eq. **4**. Additionally, the variable t in the expression is used to adjust the size of the adaptive window and is selected from a candidate set T, such as $T=\left\{5,10,15,\cdots ,100\right\}$. This measurement of regional association can be conducted on each SNP along a given chromosome. To reduce the computational cost while preserving essential information, we use *pivotal SNPs* selected at regular intervals along the chromosome, for instance, at indices such as $I=\left\{1,11,21,\cdots \right\},$ representing every 10th SNP.

As a remark, the estimated effect size $\hat{\beta }$, used in Eq. **4**, should ideally be obtained from a separate dataset. In cases where a separate dataset is unavailable, as we assume for this study, we implement a data-splitting procedure: Approximately half of the subject samples are randomly set aside to estimate the effect size using a standard GWAS procedure. While this approach is supported by the existing literature (23, 46) in the context of PRS calculations, considering more refined methodologies, such as the cross-validation approach, could yield further improvements and is worth further exploration. A detailed discussion along this line is provided in *SI Appendix*, Section S.6.2. To reduce the variance of the calculated RAS due to the uncertainty in the estimated effect size $\hat{\beta },$ such data-splitting procedure is repeated multiple times. The mean RAS from these repetitions is then computed for each individual SNP to produce the final results.

It is noteworthy that if the SNP set used in Eq. **4** covers the entire genome, our introduced LPRS is reduced to the PRS, which is commonly used as an estimate of an individual’s genetic liability to a trait or a disease across various biomedical research (21–23). However, unlike the PRS, our method employs estimated effect sizes in a more versatile manner, emphasizing local associations rather than the overall genetic association. This is why PRS is useful for estimating the overall heritability, but our LPRS can identify specific associated SNPs.

Additionally, it is critical to mention that our method requires a rigorous quality control process similar to that used in PRS calculations and traditional GWAS. This includes LD pruning, which is performed to prevent score inflation, typically using a threshold of 0.2 (37).

### Peak Point Detection.

Once the RAS for each pivotal SNP in an index set I are obtained, they can be formulated into time series data, denoted by X=(X_i: i ∈I) with each time point corresponding to a pivotal SNP from set I. These data can be visualized in a trend plot to understand how our method works. Fig. 1*B* provides an illustrative example of such a plot, based on a simulation example that was discussed in the previous section. In the plot, potential association regions are easily identifiable as noticeable peaks amid the noisy signal. A detailed theoretical explanation of this phenomenon is provided in *SI Appendix*.

For this reason, change point detection algorithms (47, 48), commonly used in time series data, are suitable for identifying peaks that represent potential association regions in the RAS-formulated trend data. Since the number of association regions is unknown, a variant of the method introduced in Zhang (49) is applied to detect significant peaks. Specifically, we consider a sliding window starting from the first pivotal SNP. Within this window, we first conduct a statistical test to determine the presence of a change point (50, 51). If the null hypothesis (no change point) is rejected, the change point finding algorithm identifies the most likely change point location.

**Fig. 5.**
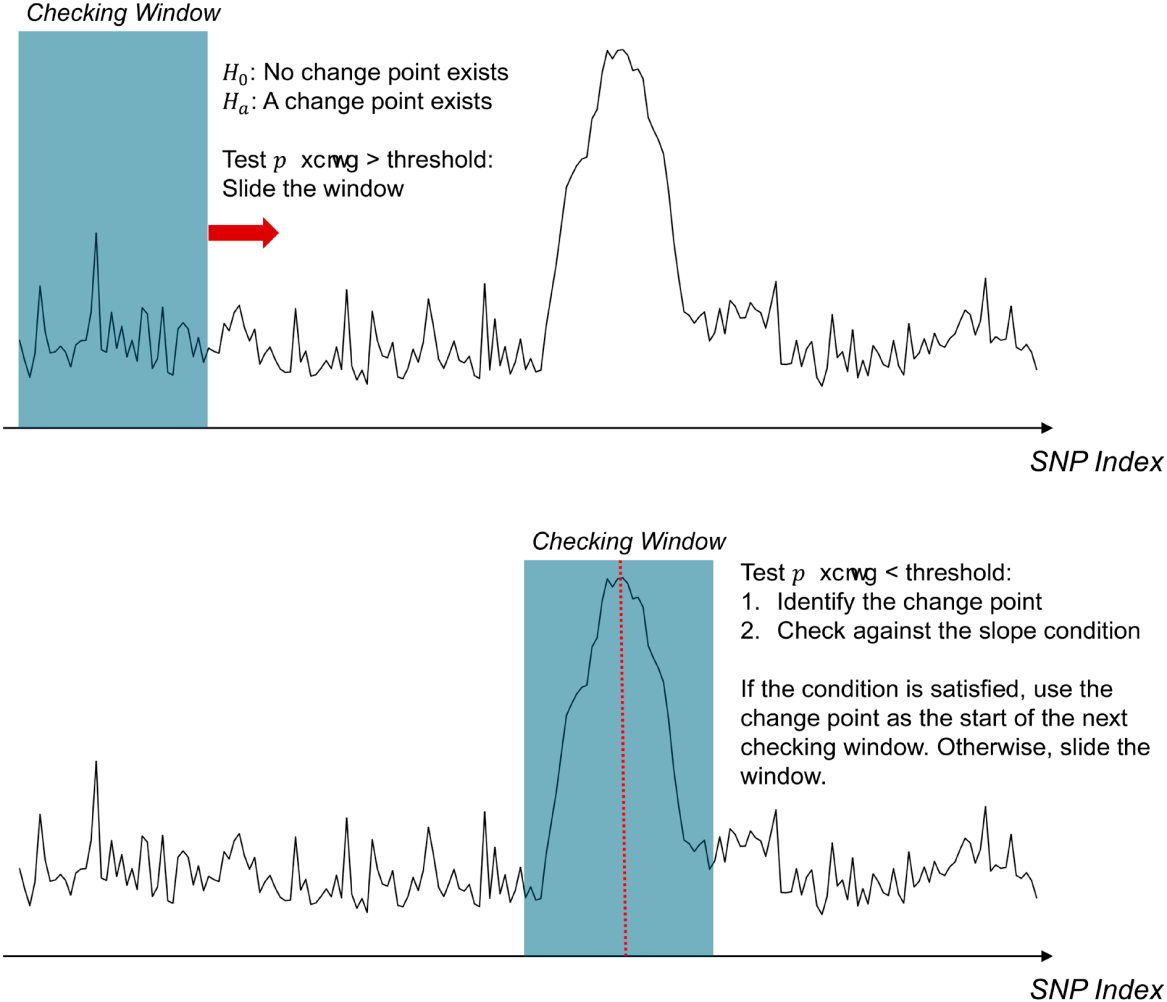
Illustration figure of the proposed peak point detection algorithm with a sliding window approach.

Subsequently, we assess whether this change point qualifies as a peak by verifying that the left slope is significantly greater than zero and the right slope is significantly less than zero. If these conditions are met, the identified change point serves as the starting point for the next window; otherwise, the window shifts to the next position for further testing. In our implementation, we ensure a substantial (e.g., 90% of the window size) overlap between adjacent windows. This configuration effectively addresses potential issues that arise when causal variants are located near the borders of the windows. This process continues until the window reaches the last pivotal SNP. Fig. 5 graphically illustrates this procedure. As the final step in our algorithm, detected peak points are filtered based on a preset magnitude threshold to confirm the association regions. This threshold also helps control the type I error rate of the detection. A detailed implementation of the proposed method is provided in *SI Appendix*.

## Supplementary Material

Appendix 01 (PDF)

Code S01 (TXT)

Code S02 (TXT)

Code S03 (TXT)

## Data Availability

The data used in this study are available from the Adolescent Brain Cognitive Development (ABCD) study® (24, 25). These data were accessed through the ABCD Data Repository. Access to the ABCD data requires an approved Data Use Agreement (DUA) and is contingent upon adherence to the study’s data use policies and protocols. The specific dataset used in this study correspond to the ABCD study release 5.0. UK Biobank data (43) are used in the supporting information and publicly available with permission at https://www.ukbiobank.ac.uk/. We developed the software to perform GWAS using RAS and made it available to the public (52).
