# Supplementary material for: Empowering genome-wide association studies via a visualizable test based on the regional association score
Source: Proc Natl Acad Sci U S A. 2025 Feb 25;122(9):e2419721122. doi: 10.1073/pnas.2419721122 (PMC11892588; doi:10.1073/pnas.2419721122)
Supplement: Supplementary file 1 — Appendix 01 (PDF) [file pnas.2419721122.sapp.pdf]

Empowering Genome-wide Association Studies via a Visualizable Test Based on Regional Association Score :

Supporting Information

Yiran Jiang and Heping Zhang
Department of Biostatistics, Yale University

Supplementary Text

S.1 Theoretical Results

S.1.1 Theoretical Explanation of the RAS Method

In this Section, we provide a theoretical understanding of the proposed RAS method in estimating regional association.

For the convenience of presentation and without loss of generality, consider the phenotype to be continuous. Denote the SNP matrix used to estimate the effect size β to be $\tilde{\mathbf{G}}_{\tilde{n}\times p} = (\tilde{\mathbf{g}}_1, \ldots, \tilde{\mathbf{g}}_p)'$ with $(\tilde{\mathbf{g}}_j)_{\tilde{n}\times 1}$ be the observed vector for the SNP j, $j = 1, \ldots, p$. Denote the corresponding phenotype vector to be $\tilde{\mathbf{y}}_{\tilde{n}\times 1}$. Assume $\tilde{\mathbf{y}}$ is generated by the regression model

$$\tilde{\mathbf{y}} = \tilde{\mathbf{G}}\beta + \tilde{\boldsymbol{\epsilon}}, \quad \tilde{\boldsymbol{\epsilon}} \sim N(0, \sigma^2)$$

with unknown $\beta_{p\times 1} = (\beta_1, \ldots, \beta_p)'$ and σ. Calculating the estimated effect size requires regressing $\tilde{\mathbf{y}}$ on each single SNP j, $j = 1, \ldots, p$. The estimated weight (or effect size) takes

the form

$$\begin{aligned}\hat{\beta}_j &= \frac{\tilde{\mathbf{g}}_j^T \tilde{\mathbf{y}}}{\tilde{\mathbf{g}}_j^T \tilde{\mathbf{g}}_j} \\ &= \frac{\tilde{\mathbf{g}}_j^T (\tilde{\mathbf{g}}_j \beta_j + \tilde{\mathbf{G}}_{-j}\beta_{-j} + \tilde{\boldsymbol{\epsilon}})}{\tilde{\mathbf{g}}_j^T \tilde{\mathbf{g}}_j} \\ &= \beta_j + \frac{\tilde{\mathbf{g}}_j^T \left(\tilde{\mathbf{G}}_{-j}\beta_{-j} + \tilde{\boldsymbol{\epsilon}}\right)}{\tilde{\mathbf{g}}_j^T \tilde{\mathbf{g}}_j} \\ &= \beta_j + \frac{\tilde{\mathbf{g}}_j^T \left(\tilde{\mathbf{y}} - \tilde{\mathbf{g}}_j\beta_j\right)}{\tilde{\mathbf{g}}_j^T \tilde{\mathbf{g}}_j} \\ &:= \beta_j + \beta_{\tilde{\mathbf{y}} - \tilde{\mathbf{g}}_j\beta_j | \tilde{\mathbf{g}}_j}\end{aligned} \tag{S1}$$

where $\tilde{\mathbf{G}}_{-j}$ denotes the SNP matrix without column j and β_{-j} the true coefficient vector without element j. The term $\beta_{\tilde{\mathbf{y}} - \tilde{\mathbf{g}}_j\beta_j | \tilde{\mathbf{g}}_j}$ in the last line in (S1) denotes the obtained coefficient by regressing the adjusted response vector $\tilde{\mathbf{y}} - \tilde{\mathbf{g}}_j\beta_j$, which removes the effect of the j-th SNP, on the observed j-th SNP vector $\tilde{\mathbf{g}}_j$. We further notice that

$$\begin{aligned}\beta_{\tilde{\mathbf{y}} - \tilde{\mathbf{g}}_j\beta_j | \tilde{\mathbf{g}}_j} &= \frac{\tilde{\mathbf{g}}_j^T \left(\tilde{\mathbf{G}}_{-j}\beta_{-j} + \tilde{\boldsymbol{\epsilon}}\right)}{\tilde{\mathbf{g}}_j^T \tilde{\mathbf{g}}_j} \\ &= \frac{\tilde{\mathbf{g}}_j^T \tilde{\mathbf{G}}_{-j}\beta_{-j} + \tilde{\mathbf{g}}_j^T \tilde{\boldsymbol{\epsilon}}}{\tilde{\mathbf{g}}_j^T \tilde{\mathbf{g}}_j}.\end{aligned} \tag{S2}$$

Since $\tilde{\mathbf{g}}_j^T \tilde{\boldsymbol{\epsilon}} \to 0$ as $\tilde{n} \to \infty$ by assumption, it can be seen that the term $\tilde{\mathbf{y}} - \tilde{\mathbf{g}}_j\beta_j$ vanishes asymptotically if $\beta_{-j} = \mathbf{0}$ or $\tilde{\mathbf{g}}_j^T \tilde{\mathbf{G}}_{-j} = \mathbf{0}$. Furthermore, in the context of GWAS, it is reasonable to assume $\tilde{\mathbf{G}}$ to be non-orthogonal due to the existence of linkage disequilibrium (LD). To conclude, the estimated effect size $\hat{\beta}_j$ typically deviates from the true effect size β_j, even asymptotically.

Without loss of generality, consider the true signal region to range from SNP $k = 1, \ldots, K$. That is, $\beta_k \neq 0$ if $k = 1, \ldots, K$ and $\beta_k = 0$ otherwise. Denote the SNP matrix used to analyze the regional association using the obtained estimated effect size $\hat{\beta}$ to be $\mathbf{G}_{n\times p} = (\mathbf{g}_1, \ldots, \mathbf{g}_p)'$ and the corresponding phenotype vector to be $\mathbf{y}_{n\times 1}$. The true model can be expressed as

$$\mathbf{y} = \sum_{k=1}^{K} \beta_k \mathbf{g}_k + \boldsymbol{\epsilon}, \quad \boldsymbol{\epsilon} \sim N(0, \sigma^2).$$

Now, consider the adaptive window to cover a region indexed by $1, \dots, J$. The obtained localized polygenic risk score (LPRS) vector when aggregating the estimated effect within the region can be expressed as

$$\hat{\mathbf{S}}_1^J = \sum_{j=1}^{J} \hat{\beta}_j \mathbf{g}_j = \sum_{j=1}^{J} \beta_j \mathbf{g}_j + \sum_{j=1}^{J} \beta_{\tilde{\mathbf{y}}-\tilde{\mathbf{g}}_j\beta_j|\tilde{\mathbf{g}}_j} \mathbf{g}_j$$

by (S1). The significance of $\hat{\mathbf{S}}_1^J$ when regressing $\mathbf{y}$ on $\hat{\mathbf{S}}_1^J$ is positively related to the linear relationship

$$\begin{aligned}\operatorname{cov}(\hat{\mathbf{S}}_1^J, \mathbf{y}) &= \operatorname{cov}\left(\sum_{j=1}^{J} \beta_j \mathbf{g}_j + \sum_{j=1}^{J} \beta_{\tilde{\mathbf{y}}-\tilde{\mathbf{g}}_j\beta_j|\tilde{\mathbf{g}}_j} \mathbf{g}_j, \sum_{k=1}^{K} \beta_k \mathbf{g}_k + \boldsymbol{\epsilon}\right) \\ &= \operatorname{cov}\left(\sum_{j=1}^{J} \beta_j \mathbf{g}_j, \sum_{k=1}^{K} \beta_k \mathbf{g}_k + \boldsymbol{\epsilon}\right) + \operatorname{cov}\left(\sum_{j=1}^{J} \beta_{\tilde{\mathbf{y}}-\tilde{\mathbf{g}}_j\beta_j|\tilde{\mathbf{g}}_j} \mathbf{g}_j, \sum_{k=1}^{K} \beta_k \mathbf{g}_k + \boldsymbol{\epsilon}\right),\end{aligned}$$

where the first term can be seen as the *signal*, and the second term plays the role of the *noise*, when $J < K$. Specifically, the signal or the significance of the $\hat{\mathbf{S}}_1^J$ is contributed by the term $\sum_{j=1}^{J} \beta_j \mathbf{g}_j$, which serves as a part of the true model $\sum_{k=1}^{K} \beta_k \mathbf{g}_k + \boldsymbol{\epsilon}$. On the other hand, the noise is given by the term $\sum_{j=1}^{J} \beta_{\tilde{\mathbf{y}}-\tilde{\mathbf{g}}_j\beta_j|\tilde{\mathbf{g}}_j} \mathbf{g}_j$, as it does not provide additional information to the unexplained components $\sum_{j=J+1}^{K} \beta_j \mathbf{g}_j$.

Next, consider the adaptive window to increase its size to cover an additional SNP positioned at the index $J+1$. Aggregating the LPRS from J to $J+1$ updates the linear relationship strength to

$$\begin{aligned}\operatorname{cov}(\hat{\mathbf{S}}_1^{J+1}, \mathbf{y}) &= \operatorname{cov}(\hat{\mathbf{S}}_1^J, \mathbf{y}) + \operatorname{cov}\left(\beta_{j+1}\mathbf{g}_{j+1} + \beta_{\tilde{\mathbf{y}}-\tilde{\mathbf{g}}_{j+1}\beta_{j+1}|\tilde{\mathbf{g}}_{j+1}} \mathbf{g}_{j+1}, \sum_{k=1}^{K} \beta_k \mathbf{g}_k + \boldsymbol{\epsilon}\right) \\ &= \operatorname{cov}(\hat{\mathbf{S}}_1^J, \mathbf{y}) + \operatorname{cov}\left(\beta_{j+1}\mathbf{g}_{j+1}, \sum_{k=1}^{K} \beta_k \mathbf{g}_k + \boldsymbol{\epsilon}\right) + \operatorname{cov}\left(\beta_{\tilde{\mathbf{y}}-\tilde{\mathbf{g}}_{j+1}\beta_{j+1}|\tilde{\mathbf{g}}_{j+1}} \mathbf{g}_{j+1}, \sum_{k=1}^{K} \beta_k \mathbf{g}_k + \boldsymbol{\epsilon}\right),\end{aligned}$$

which introduces additional signals provided by the term $\beta_{j+1}\mathbf{g}_{j+1}$ and additional noise by the term $\beta_{\tilde{\mathbf{y}}-\tilde{\mathbf{g}}_{j+1}\beta_{j+1}|\tilde{\mathbf{g}}_{j+1}} \mathbf{g}_{j+1}$. However, if $J = K$, aggregating $J = K$ to $J = K+1$ gives

$$\operatorname{cov}(\hat{\mathbf{S}}_1^{K+1}, \mathbf{y}) = \operatorname{cov}(\hat{\mathbf{S}}_1^K, \mathbf{y}) + \operatorname{cov}\left(\beta_{\tilde{\mathbf{y}}-\tilde{\mathbf{g}}_{K+1}\beta_{K+1}|\tilde{\mathbf{g}}_{K+1}} \mathbf{g}_{K+1}, \sum_{k=1}^{K} \beta_k \mathbf{g}_k + \boldsymbol{\epsilon}\right)$$

as $\beta_{K+1} = 0$, which provides no additional signal but additional noise brought by the term $\beta_{\tilde{\mathbf{y}}-\tilde{\mathbf{g}}_{K+1}\beta_{K+1}|\tilde{\mathbf{g}}_{K+1}} \mathbf{g}_{K+1}$, resulting in potential reduction in the significance level.

To conclude, since the pivotal SNPs locating on the central of a true association region is likely to produce a window that can cover all true SNPs, it could result in more significant association between the calculated LPRS and the phenotypes compared to other pivotal SNPs which introduce more noise. As a result, the p-value trend plot will appear as peaks at the vicinity of these pivotal SNPs.

S.1.2 Theoretical Explanation for the Reduced Type I Error When True Signal is Present

In this section, we provide theoretical insights into how our application of changepoint detection algorithm has the potential to reduce the risk of inflated type I error rates in genetic association studies.

Consider a scenario where we are analyzing the association between a phenotype $\mathbf{y}$ and genotype data, represented by the matrix $\mathbf{G}_{n\times p} = (\mathbf{g}_1, \ldots, \mathbf{g}_p)'$. Each column of the genotype data is standardized to have a mean of 0 and a variance of 1. Let's consider three variants indexed by $1, 2, 3$ across a chromosome, located at positions $t_1 < t_2 < t_3$. Variant 2 is in linkage disequilibrium (LD) with variants 1 and 3. We denote the Pearson correlations as $\rho_{12} = \text{corr}(G_1, G_2)$ and $\rho_{23} = \text{corr}(G_2, G_3)$, with $0 < \rho_{12}, \rho_{23} < 1$. The true association model is given by:

$$Y = \beta_2 G_2 + \epsilon, \quad \epsilon \sim N(0, \sigma^2),$$

where $\beta_2 \neq 0$ represents the effect size, and σ is the standard error. This suggests that the true signal comes from variant 2 but not from variants 1 and 3. Consider conducting three separate association tests:

$$Y \sim G_1, \quad Y \sim G_2, \quad Y \sim G_3$$

Denote the obtained test statistics by Z_1, Z_2, Z_3 with corresponding p-values p_1, p_2, p_3. We present Proposition S1 below, suggesting that the canonical association tests will

asymptotically result in type I error as $n \to \infty$ when LD is present between the tested variants.

Proposition S1. *Under the stated conditions, $p_1,\ p_2,\ p_3\ \to 0$ as $n \to \infty$.*

Proof. For Z_2, the test statistic is given by:

$$Z_2 \;=\; \frac{\widehat{\beta}_2}{\mathrm{SE}(\widehat{\beta}_2)} \;\sim\; \sqrt{n}\beta_2,$$

and consequently,

$$p_2 \;=\; 2\big[1 - \Phi(|Z_2|)\big] \;\to\; 0$$

as $n \to \infty$, where $\Phi(\cdot)$ denotes the cumulative distribution function (CDF) of the standard normal distribution.

For G_1, under the true model $Y = \beta_2 G_2 + \epsilon$, the least-squares estimate of β_1 in the regression of Y on G_1 is, for large n,

$$\begin{aligned}\widehat{\beta}_1 \;\approx\; \frac{\mathrm{Cov}(Y,\, G_1)}{\mathrm{Var}(G_1)} \;&=\; \frac{\mathrm{Cov}(\beta_2\, G_2,\ G_1)}{\mathrm{Var}(G_1)} \\ &= \beta_2\, \frac{\mathrm{Cov}(G_1,\, G_2)}{\mathrm{Var}(G_2)} \\ &= \beta_2 \rho_{12},\end{aligned}$$

since $\widehat{\beta}_1$ essentially estimates the indirect effect of G_2 on Y mediated by the correlation between G_1 and G_2. As n increases, the standard error of $\widehat{\beta}_1$ decreases, on the order of $1/\sqrt{n}$, leading to:

$$Z_1 \;=\; \frac{\widehat{\beta}_1}{\mathrm{SE}(\widehat{\beta}_1)} \;\sim\; \sqrt{n}\sigma\beta_2\rho_{12} \to \infty,$$

Hence,

$$p_1 \;=\; 2\big[1 - \Phi(|Z_1|)\big] \to 0.$$

Applying a similar argument for G_3, we conclude that $p_3 \to \infty$.

□

In Proposition S2, we demonstrate that the convergence rates of the p-values for these variants differ significantly.

Proposition S2. *Under the stated conditions, $p_2/p_1 \to 0$ and $p_2/p_3 \to 0$ as $n \to \infty$.*

Proof. Given the exponential decay behavior of the tail probabilities for normal CDF (see Baricz, 2008, and references therein),

$$\begin{aligned} p_1 &\sim \exp\left(-\frac{Z_1^2}{2}\right) = \exp\left(-\frac{n\left(\beta_2\rho_{12}\right)^2}{2}\right) \\ p_2 &\sim \exp\left(-\frac{Z_2^2}{2}\right) = \exp\left(-\frac{n\beta_2^2}{2}\right) \\ p_3 &\sim \exp\left(-\frac{Z_3^2}{2}\right) = \exp\left(-\frac{n\left(\beta_2\rho_{23}\right)^2}{2}\right) \end{aligned}$$

Consequently,

$$\begin{aligned} \frac{p_2}{p_1} &\sim \exp\left(-\frac{n}{2}\left[\beta_2^2 - (\beta_2\rho_{12})^2\right]\right) = \exp\left(-\frac{n}{2}\beta_2^2\left(1-\rho_{12}\right)\right) \\ \frac{p_2}{p_3} &\sim \exp\left(-\frac{n}{2}\left[\beta_2^2 - (\beta_2\rho_{23})^2\right]\right) = \exp\left(-\frac{n}{2}\beta_2^2\left(1-\rho_{23}\right)\right). \end{aligned} \tag{S3}$$

Given that $\rho_{12} \in (0,1)$ and $\rho_{13} \in (0,1)$, it follows from (S3) that $p_2/p_1 \to 0$ and $p_2/p_3 \to 0$ as $n \to \infty$. □

It can be further derived from the conclusion of Proposition S2 that $(-\log p_2) - (-\log p_1) \to \infty$ and $(-\log p_2) - (-\log p_3) \to \infty$.

The insight from Proposition S1 highlights that when LD exists, canonical association tests may asymptotically lead to false discoveries in regions linked through LD to the true signal region. According to Proposition S2, the transformed p-values for the true variant asymptotically stand out as peaks, making it detectable by changepoint detection algorithms.

The major implication from this theoretical analysis is that focusing on the *relative signal strength* through changepoint detection can help mitigate the issues of false discovery that arise from assessing *absolute signal strength* in canonical association tests.

We acknowledge the limitations of our simplified theoretical analysis framework, which is based on asymptotic conditions and single variant testing. The primary goal of this theoretical analysis is to provide foundational insights. These concepts can be extended to set-based testing methods that aggregate regional information, such as LPRS and SKAT, where the fundamental insights remain applicable. Importantly, our RAS method implicitly assumes that "not all variants on the chromosome are significant", which is a practical assumption but requires further theoretical elaboration. More comprehensive analysis in this area is necessary and merits additional exploration.

S.2 Implementation Details

In the simulation study, we use the R package `SKAT` (Lee et al., 2023) for conducting the alternative methods burden tests, SKAT, SKAT-O and RC-SKAT. We use the R package `CauchyGM` (Kim, 2025) to conduct the alternative methods CauchyGM and CauchyGM-O.

R is also used for the implementation of our proposed method. The proposed method is conducted in details as follows.

S.2.1 RAS Calculation

Regarding the proposed RAS calculated as

$$RAS_j = \max_{t \in \mathcal{T}} f\left(\sum_{k=\max(j-t,1)}^{\min(j+t,p)} \hat{\beta}_k \mathbf{g}_k, \mathbf{y}\right), \tag{S4}$$

the window size $\mathcal{T}$ is chosen to span from 5 to 100. The pivotal SNPs are selected at regular intervals of 10 SNPs. That is, the pivotal SNPs have indices $I = \{1, 11, 21, \ldots\}$. The data-splitting procedure for calculating the estimated effect size as well as the RAS is repeated for 5 times, and the mean RAS from these repetitions is then computed for each individual to produce the final results.

S.2.2 Peak Point Detection

The sliding window size used for change point detection is 100, and the Davies test (Davies, 1987) is used for testing the existence of a change point. The p-value threshold value used in the simulation study and the real data analysis is 10^{-8}. The test for left slope being greater than 0 and right slope being smaller than 0 uses the p-value threshold value of 10^{-10}, with the window size of 30. Two additional tests for verifying the peak condition is conducted by testing the existence of the change point within the window of size 100, starting from the detected change point to left and right side. In the simulation study and real data analysis, the magnitude threshold of RAS for the peak point is set as 2.5. The

detected point is finally identified as the peak point if either test gives the p-value of less than 10^{-15}. The package `segmented` (Muggeo, 2008) is used for implementing the proposed peak point detection algorithm.

S.3 Additional Illustrative Plots

The visualization of the association region detection results with the proposed RAS method using the simulation examples given in the paper are provided in Figure S3 and S4.

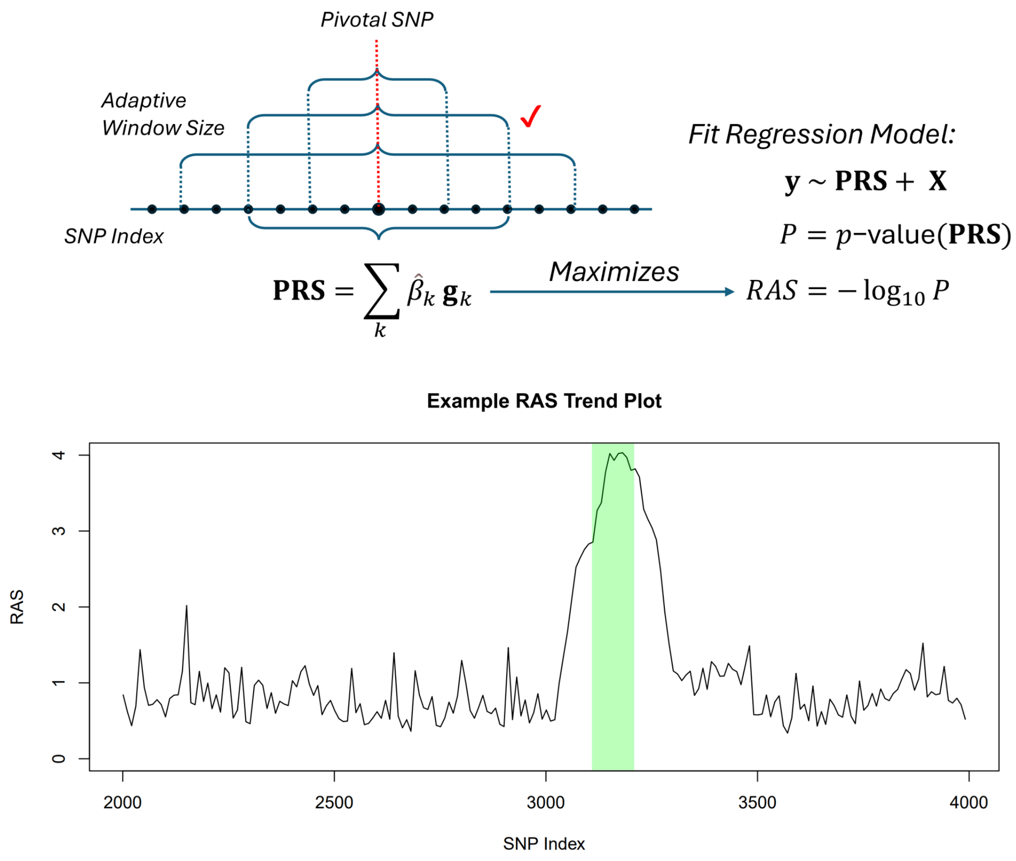

Figure S1: Graphical illustration of the process for calculating the RAS for each individual pivotal SNP using an adaptive window. The bottom plot is an example trend plot generated by the RAS results obtained from a simulation study with ABCD data. The green semi-transparent time window represents the true association region

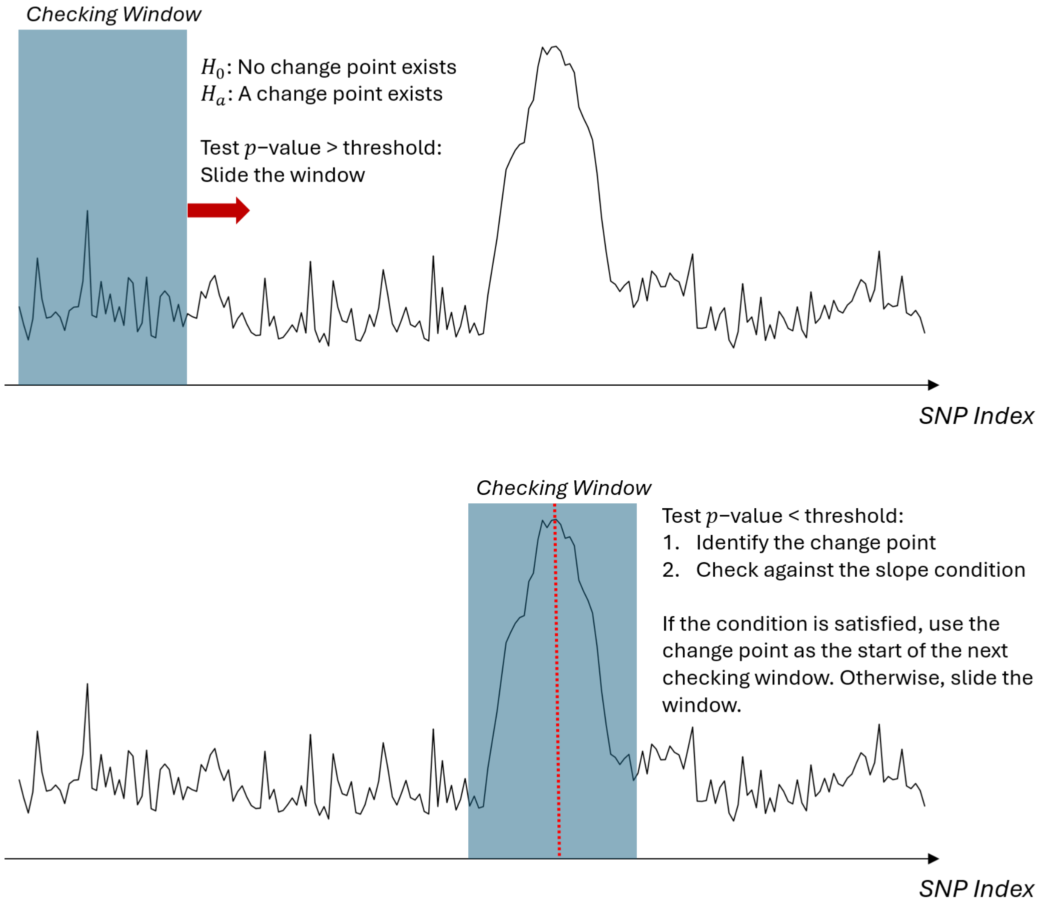

Figure S2: Illustration figure of the proposed peak point detection algorithm with a sliding window approach.

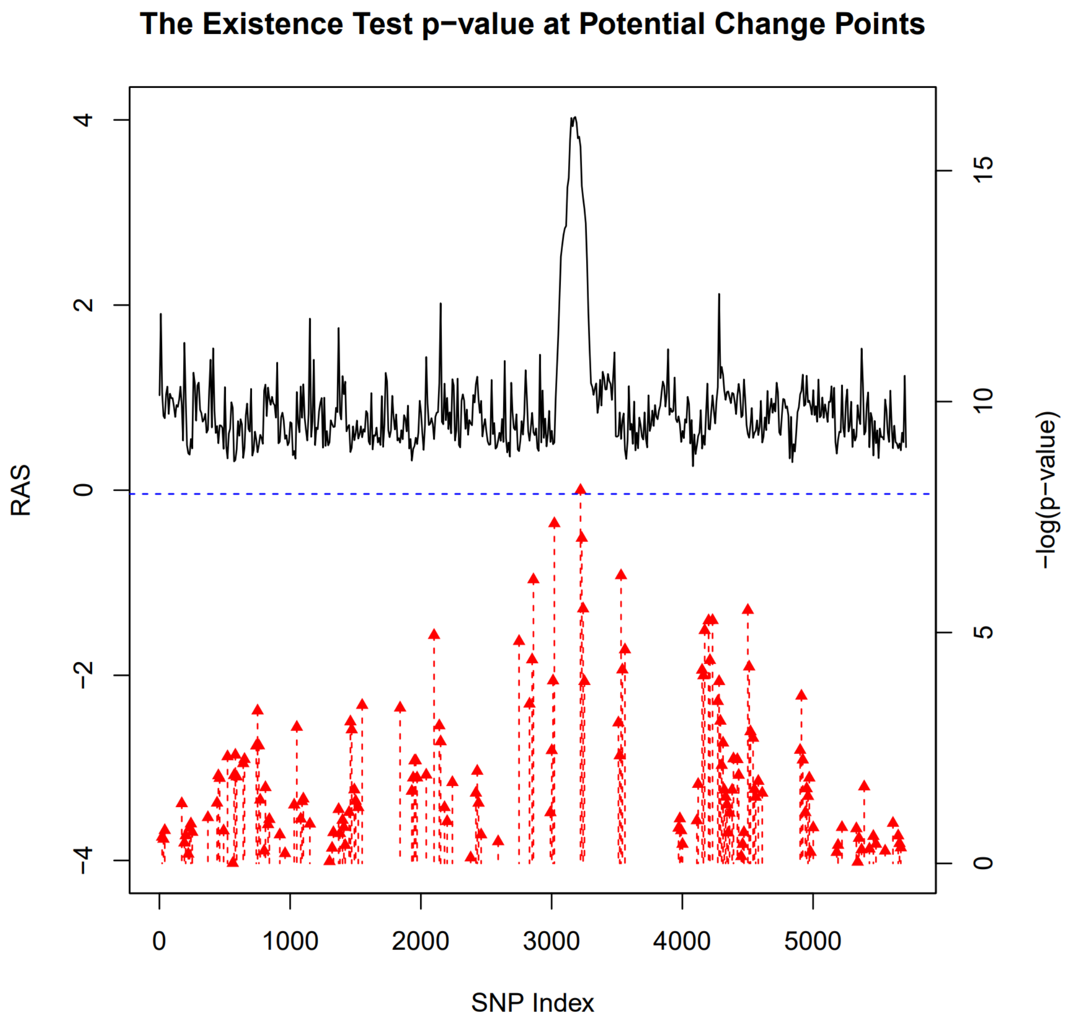

Figure S3: Results from the sliding window peak point detection algorithm using the illustrative example ($M = 1$). Red dotted lines indicate change points identified in certain sliding windows. Red symbols represent the p-value of the test for the existence of a change point within the corresponding sliding window. The blue dotted horizontal line signifies the threshold value of the test. The change points that fail to meet the slope condition are not shown in the plot.

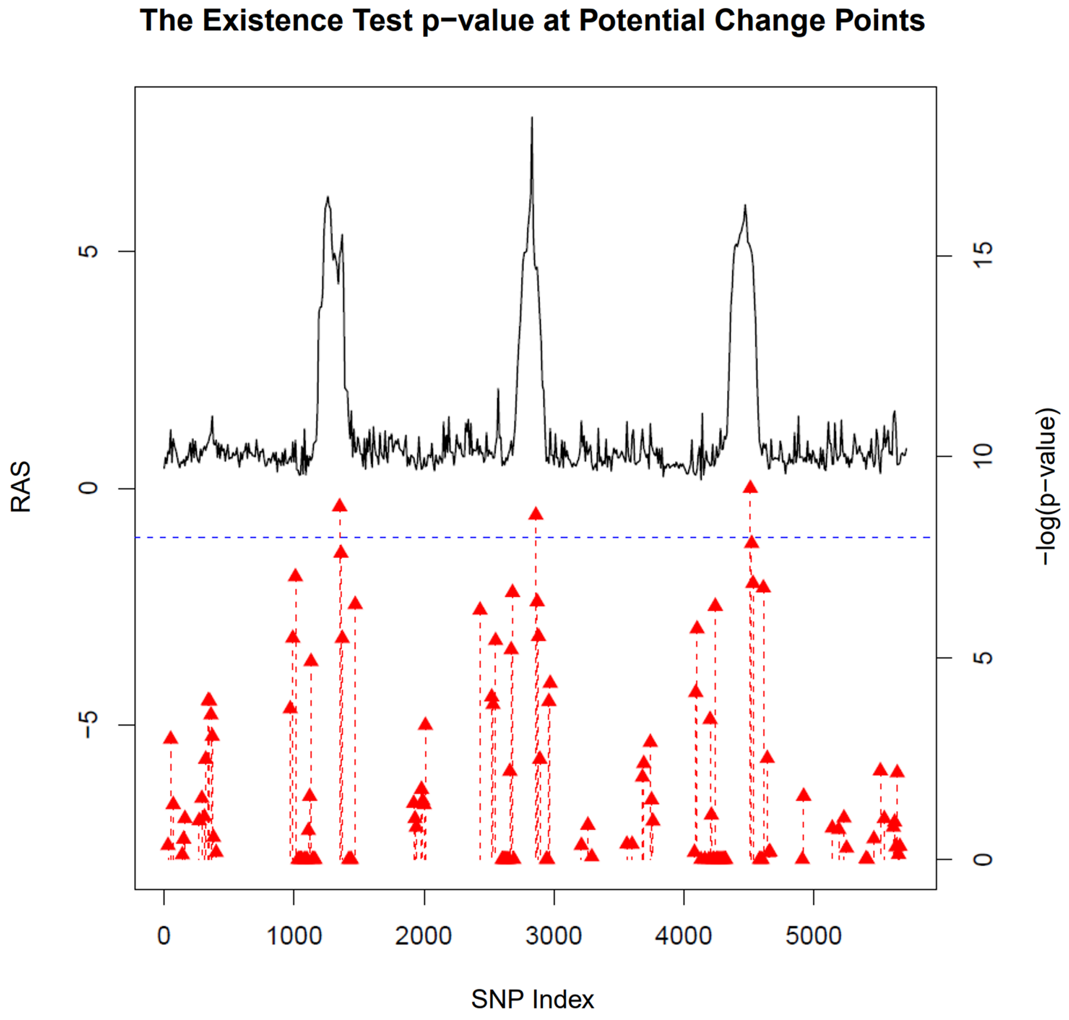

Figure S4: Results from the sliding window peak point detection algorithm using another illustrative example with $M = 3$. The interpretation of the plot is the same as Figure S3.

S.4 Additional Information Regarding the ABCD Data

S.4.1 Distribution of Minor Allele Frequency (MAF)

We present the distribution of MAF in the genotyping array data from the ABCD study. The distribution of MAF across Chromosome 21 is shown in Figure S5. The distribution of MAF across the entire genome is shown in Figure S6. For both Chromosome 21 and the entire genome, the percentage of the low-frequency variants ($0.01 \leq \text{MAF} < 0.05$) and the common variants $\text{MAF} \geq 0.05$ are 20% and 80%, correspondingly.

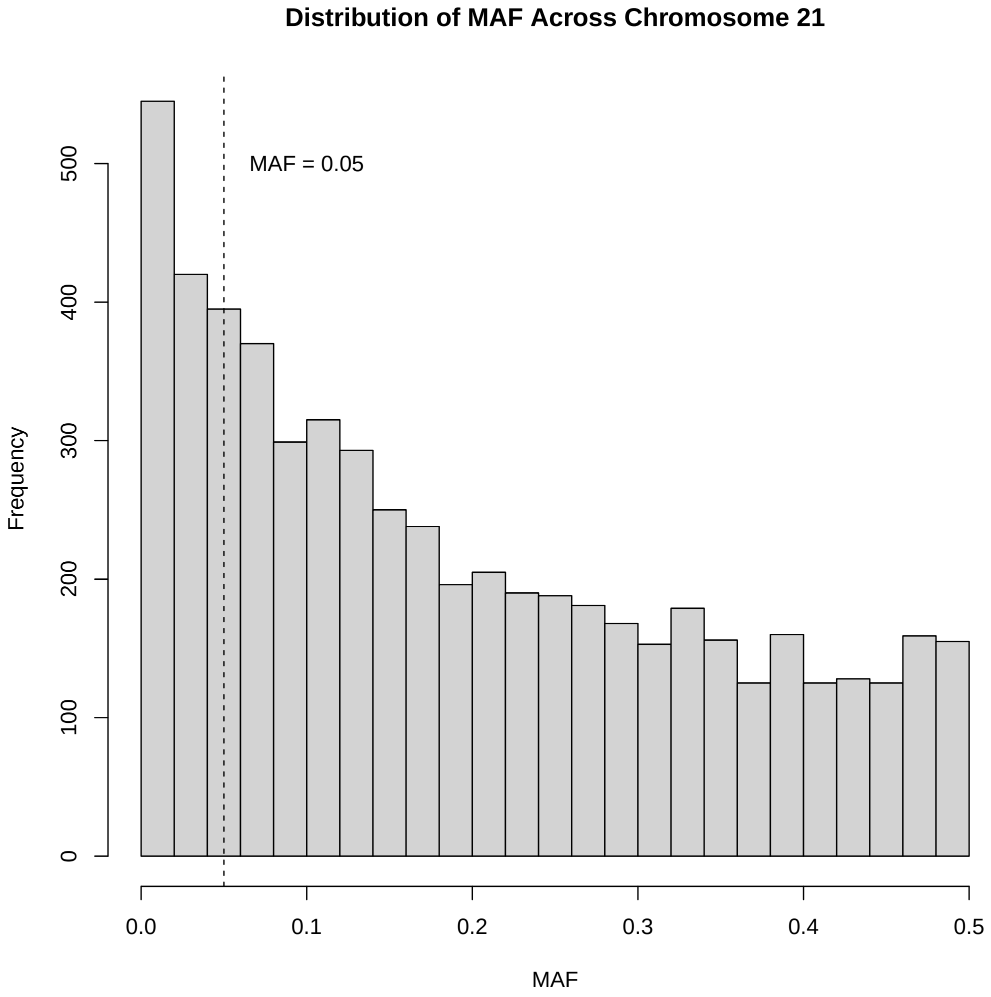

Figure S5: Histogram of MAF across Chromosome 21 in the ABCD study genotype data. The vertical dotted line represents a MAF of 0.05, marking the threshold between low-frequency variants and common variants.

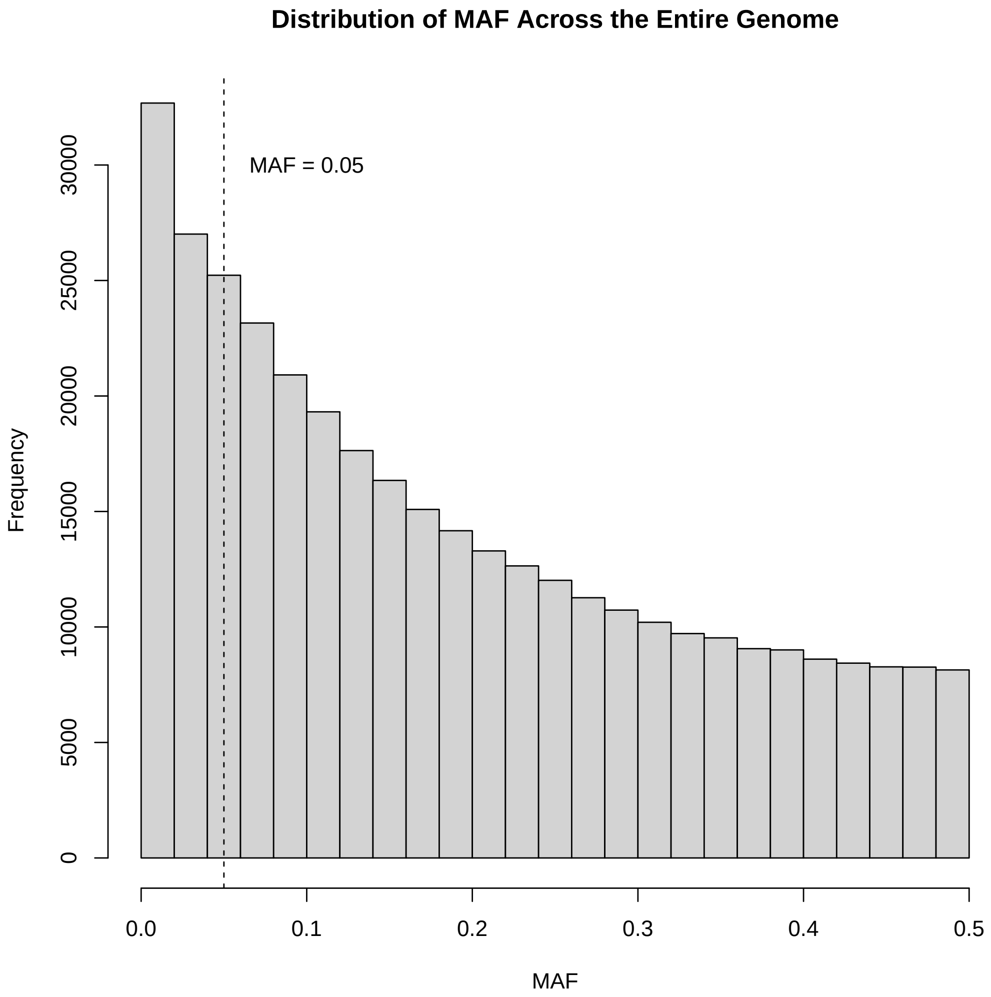

Figure S6: Histogram of MAF across the entire genome in the ABCD study genotype data. The vertical dotted line represents a MAF of 0.05, marking the threshold between low-frequency variants and common variants.

S.4.2 Distribution of Age

Figure S7 shows the age distribution of the cohort used in the real data analysis with the ABCD dataset.

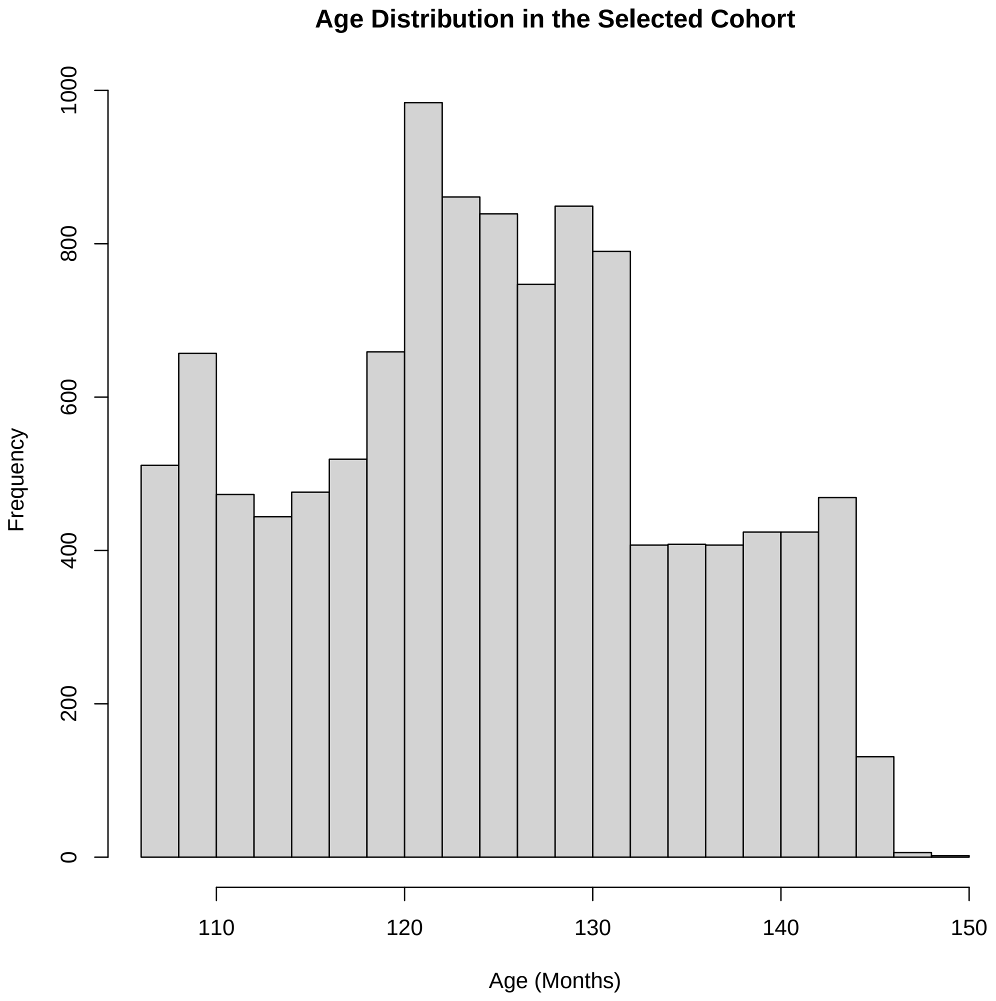

Figure S7: Histogram of age (in months) for the cohort comprising 11,488 individuals from the ABCD dataset.

S.5 Additional Discussion on Computational Efficiency

S.5.1 Scalability of the RAS Method

Our simulation study, as reported in the main manuscript, shows that our method requires a similar amount of computation time per repetition compared with existing set-based testing methods. Using a sliding window approach for calculating the LPRS and detecting change points, the computational cost increases linearly with the size of the SNP data and the sample size, under constant computational settings. This scalability demonstrates our method's applicability to large genomic datasets.

S.5.2 Trade-off Between Computational Accuracy and Burden

There is an inherent trade-off between computational accuracy and burden in our method and other existing methods. For instance, refining the sliding window approach used in set-based testing methods like SKAT to start at every single SNP could allow for denser scanning. However, such refinement becomes computationally prohibitive with large genomic datasets and typically offers negligible gains. Similarly, our RAS method includes several settings that are adjustable and influence computational efficiency:

- Candidate set of the window size used to find the optimal window size in order to calculate the LPRS;
- Density of the pivotal SNPs;
- Number of repetitions for data-splitting;
- Density of the sliding window.

Empirical experiments suggest that the settings described in the main manuscript, and detailed in Section S.2.1, yield sufficiently accurate results. Nonetheless, further evaluation along these lines is necessary.

S.5.3 Practical Guidelines

Based on the computational settings detailed in the main manuscript and summarized in Section S.2.1, our empirical data using an average CPU achieves a processing speed of approximately 3,000 SNPs per minute for continuous traits and 1,000 SNPs per minute for dichotomous cases. This speed decreases when complex covariate data are included. The linear relationship between settings and computational time allows for straightforward adjustments based on available computational resources. By modifying the parameters, one can easily calculate the estimated computational time with simple multiplications to better suit their research needs.

Regarding these parameter settings, to save computational resources, the number of repetitions for data-splitting can be reduced to a single split when the sample size is sufficiently large. This approach is supported by literature, such as Choi et al. (2020) and Dudbridge (2013), which recognizes the efficiency of a single split in the context of traditional PRS calculations.

S.6 Possible Extensions of the RAS Method

S.6.1 Extension of the RAS Method to the Transcriptome-wide association studies (TWAS)

Review of TWAS

TWAS is a gene-prioritization approach that detects trait-associated genes regulated by significant variants, primarily SNP identified from GWAS (Mai et al., 2023). This method has been increasingly recognized for its ability to enhance the power of GWAS through the integration of transcriptomic data (Ardlie et al., 2015; Cao et al., 2021; Gusev et al., 2016; Mai et al., 2023; Xu et al., 2017). According to Cao et al. (2021),

> The key insight of TWAS is that transcriptomic data can be used to select for genetic variants that are critical to gene expression (i.e. eQTLs) and which improve the quality of downstream GWAS. By modeling the association between linear combinations of variants and gene expression, TWAS effectively aggregates many genetic variants into a small number of meaningful linear combinations.

In essence, TWAS builds on the foundation laid by GWAS, offering more interpretable, efficient, tissue-specific, and powerful genetic analysis tools that are particularly suited to understanding complex traits and diseases (Mai et al., 2023).

Extension of the RAS Method

Several studies have sought to connect the set-based method such as SKAT, with TWAS. Notably, Gamazon et al. (2015) pioneered the linkage between SKAT and TWAS, with Cao et al. (2021) further advancing this concept by proposing a new methodology tailored for TWAS applications based on SKAT. We explore a potential extension of our methods to TWAS, drawing upon the ideas presented in Cao et al. (2021), though further exploration

is indeed necessary.

For the standard TWAS procedure, as outlined by Gamazon et al. (2015) and Cao et al. (2021), the gene expression versus genotype model is represented as follows:

$$Z \sim \sum \beta_i G_i + \epsilon \tag{S5}$$

where Z denotes the gene expression, G denotes the genotype, and β_i are regression parameters to be trained. Various methods for training this predictive model are discussed by Gusev et al. (2016). Then, we use the predicted gene expression $\hat{Z}$ on the data set as a substitute of the genotype data G to associate with the phenotype. The canonical model (Cao et al., 2021) is expressed as:

$$Y \sim \hat{Z} + \epsilon.$$

Here, Y denotes the phenotype data. Cao et al. (2021) uses β from model (S5) to construct the kernel K for SKAT, which is used weight the variants when testing the association between Y and X. It is important to note that standard SKAT typically assigns equal weight to all genetic variants in a region when no prior information is available.

Furthermore, as also supported by Xu et al. (2017), technically, the integration of gene-expression data offers a way to apply weights to original genotype scores, with weights provided by model (S5). Our RAS method can be extended to incorporate the weights provided by gene-expression data. A direct application is to aggregate local information of genotypes using *weighted LPRS*, where weights for each SNP are derived from β in the model (S5). This modification aims to enhance the method's sensitivity by adjusting the influence of specific genotypic information based on its regulatory impact on gene expression. The potential effectiveness and viability of these adaptations, as well as alternative approaches, warrant further investigation.

S.6.2 Discussion on Alternative Methods in Calculating PRS

Justification of the 50-50 Split Ratio

In our study, we employ a 50-50 split ratio for data splitting, a method well-supported by existing literature. The debate around the optimal procedure for data splitting in PRS calculations remains open. However, the 50-50 split is a commonly accepted approach within the field and is backed by several studies (Choi et al., 2020; Janssens, 2019; Zhao et al., 2024). Dudbridge (2013) provides substantial evidence supporting this choice:

> When a sample is to be split into two subsets, a roughly even split yields the greatest power for testing association of the score.

Further evidence from simulation studies by Dudbridge (2013) suggests that a single 50-50 split is often more effective than 10-fold cross-validation, particularly when the sample size is limited. This is corroborated by other studies which indicate that more intricate methods like 10-fold cross-validation might not always provide additional benefits and can be less effective in specific contexts (Machiela et al., 2011; Simonson et al., 2011).

Potential for Future Exploration

While we have adopted the 50-50 split ratio based on its simplicity and established efficacy, we recognize the importance of exploring other data-splitting methods for calculating PRS. The leave-one-out split and the 10-fold cross-validation approach are promising directions for future research. These methods might offer better outcomes, especially in larger sample settings, as suggested by Dudbridge (2013).

Exploring these alternatives could potentially enhance computational efficiency and improve the accuracy of our calculations. Given that our LPRS methodology shares similarities with PRS calculations, advancements in PRS can be directly applicable to LPRS, facilitating continuous improvement.

S.7 Additional Application Example: The UK Biobank Data

We have conducted an additional application study of our method to UK biobank data (`https://www.ukbiobank.ac.uk/`). The UK Biobank is a large-scale population-based cohort study that includes data from more than 500,000 individuals aged 40 to 69, recruited from across the UK (Bycroft et al., 2018). It offers a wealth of genetic and health-related information, including genetic data, medical imaging, and environmental metrics. For this study, we used imputed genomic data from the UK Biobank (Field ID: 22828) as genotype data.

We focused on Major Depressive Disorder (MDD), a binary trait, for this study. After quality control, our cohort consists of 339,085 participants (53.6% female), where 23,098 participants are cases, and 315,987 are controls. The majority of the UK Biobank population is White, offering less racial diversity compared to the ABCD data.

Previous research has identified significant variants on Chromosome 6, 10 and 11 (Yue Hu, 2025). This guided our application of the proposed RAS method to scan these chromosomes for potential association regions. The regions detected on chromosomes 10, 11 are visualized in Figure S8 and Figure S9 correspondingly. The detected regions belong to gene *NRG3* and *OOSP1*. The association between *NRG3*, a neural-enriched member of the neuregulin protein family, has been well-established in the literature (Meier et al., 2013; Paterson et al., 2017). Studies have shown that gene *OOSP1* is related to lipid measurement (Al-Khelaifi et al., 2019), which are further recognized for their connections to depression, anxiety, and stress-related disorders by recent studies (Chourpiliadis et al., 2024). The information of the previous genetic association studies for the gene *OOSP1* was sourced from the GWAS knowledgebase NHGRI-EBI GWAS Catalog (`www.ebi.ac.uk/gwas`)(Sollis et al., 2023). These results demonstrate the applicability and effectiveness of our method across large-scale datasets, underscoring its utility in diverse genetic studies.

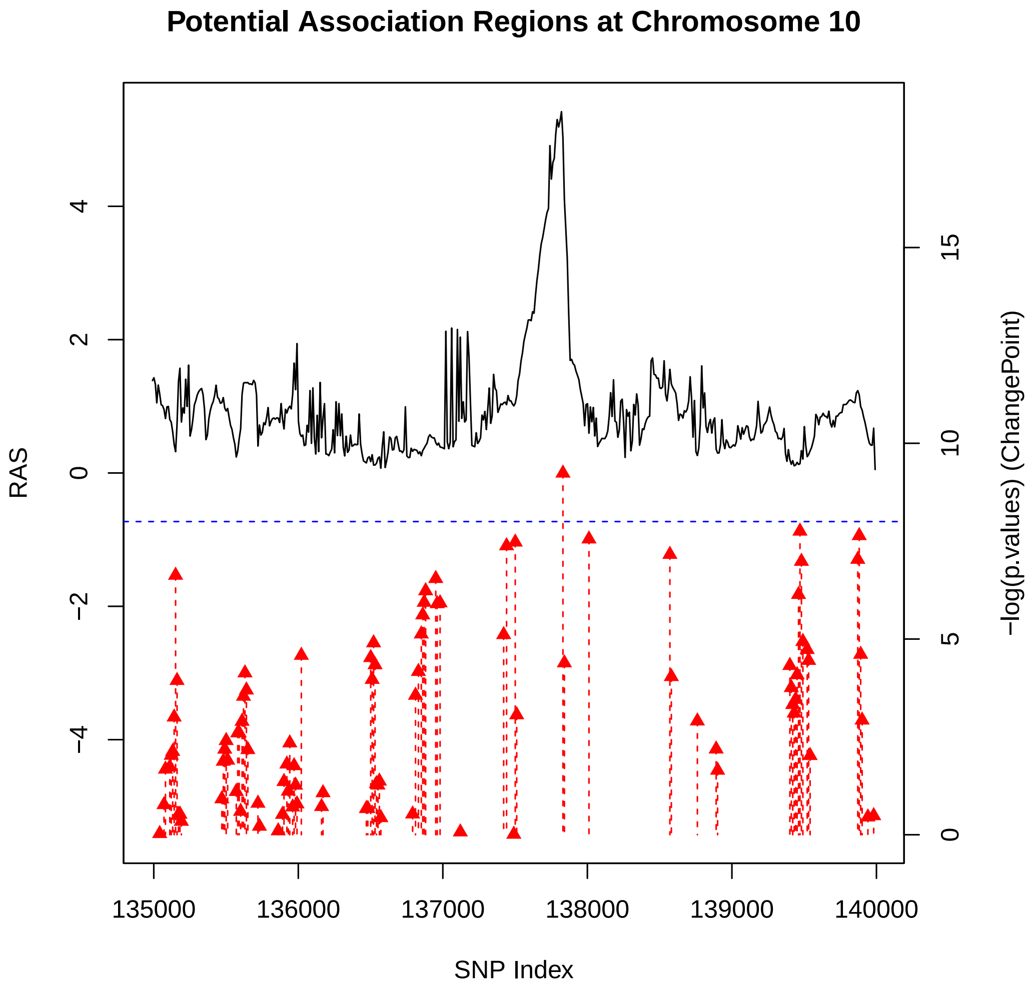

Figure S8: Detected potential association regions on chromosome 10. Red dotted lines indicate change points identified in certain sliding windows. Red symbols represent the p-value of the test for the existence of a change point within the corresponding sliding window. The blue dotted horizontal line signifies the threshold value of the test. The change points that fail to meet the slope condition are not shown in the plot.

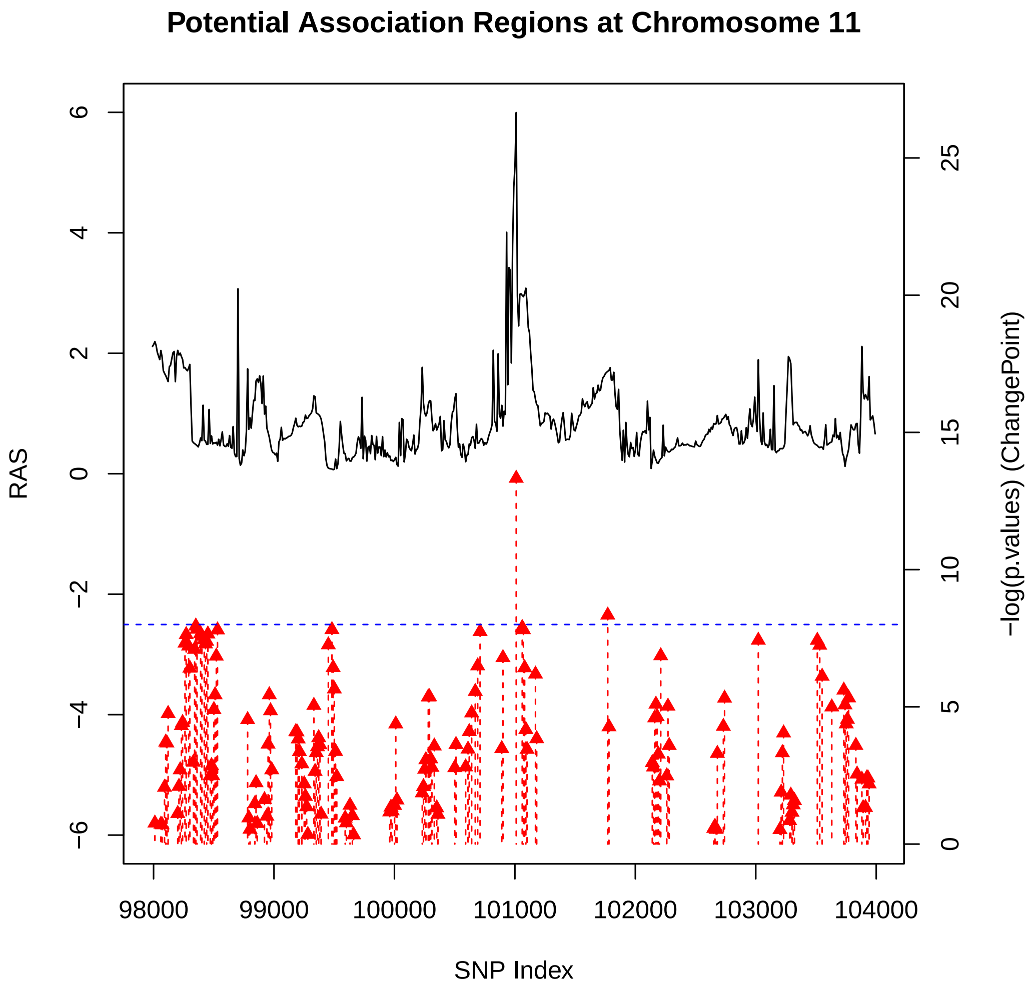

Figure S9: Detected potential association regions on chromosome 11. The interpretation of the plot is the same as Figure S8.

References

Al-Khelaifi, F., Diboun, I., Donati, F., Botrè, F., Abraham, D., Hingorani, A., Albagha, O., Georgakopoulos, C., Suhre, K., Yousri, N. A., and Elrayess, M. A. (2019). Metabolic gwas of elite athletes reveals novel genetically-influenced metabolites associated with athletic performance. *Scientific reports*, 9(1):19889–19889.

Ardlie, K. G., Deluca, D. S., Segrè, A. V., Sullivan, T. J., Young, T. R., Gelfand, E. T., Trowbridge, C. A., Maller, J. B., Tukiainen, T., Lek, M., Ward, L. D., Kheradpour, P., Iriarte, B., Meng, Y., Palmer, C. D., Esko, T., Winckler, W., Hirschhorn, J. N., Kellis, M., MacArthur, D. G., Getz, G., Shabalin, A. A., Li, G., Zhou, Y.-H., Nobel, A. B., Rusyn, I., Wright, F. A., Lappalainen, T., Ferreira, P. G., Ongen, H., Rivas, M. A., Battle, A., Mostafavi, S., Monlong, J., Sammeth, M., Mele, M., Reverter, F., Goldmann, J. M., Koller, D., Guigó, R., McCarthy, M. I., Dermitzakis, E. T., Gamazon, E. R., Im, H. K., Konkashbaev, A., Nicolae, D. L., Cox, N. J., Flutre, T., Wen, X., Stephens, M., Pritchard, J. K., Tu, Z., Zhang, B., Huang, T., Long, Q., Lin, L., Yang, J., Zhu, J., Liu, J., Brown, A., Mestichelli, B., Tidwell, D., Lo, E., Salvatore, M., Shad, S., Thomas, J. A., Lonsdale, J. T., Moser, M. T., Gillard, B. M., Karasik, E., Ramsey, K., Choi, C., Foster, B. A., Syron, J., Fleming, J., Magazine, H., Hasz, R., Walters, G. D., Bridge, J. P., Miklos, M., Sullivan, S., Barker, L. K., Traino, H. M., Mosavel, M., Siminoff, L. A., Valley, D. R., Rohrer, D. C., Jewell, S. D., Branton, P. A., Sobin, L. H., Barcus, M., Qi, L., McLean, J., Hariharan, P., Um, K. S., Wu, S., Tabor, D., Shive, C., Smith, A. M., Buia, S. A., and Consortium, T. G. (2015). The genotype-tissue expression (gtex) pilot analysis: Multitissue gene regulation in humans. *Science (American Association for the Advancement of Science)*, 348(6235):648–660.

Baricz, A. (2008). Mills' ratio: Monotonicity patterns and functional inequalities. *Journal of mathematical analysis and applications*, 340(2):1362–1370.

Bycroft, C., Freeman, C., Petkova, D., Band, G., Elliott, L. T., Sharp, K., Motyer, A., Vukcevic, D., Delaneau, O., O'Connell, J., Cortes, A., Welsh, S., Young, A., Effingham, M., McVean, G., Leslie, S., Allen, N., Donnelly, P., and Marchini, J. (2018). The uk biobank resource with deep phenotyping and genomic data. *Nature (London)*, 562(7726):203–209.

Cao, C., Kwok, D., Edie, S., Li, Q., Ding, B., Kossinna, P., Campbell, S., Wu, J., Greenberg, M., and Long, Q. (2021). ktwas: integrating kernel machine with transcriptome-wide association studies improves statistical power and reveals novel genes. *Briefings in bioinformatics*, 22(4).

Choi, S. W., Mak, T. S., and O'Reilly, P. F. (2020). Tutorial: a guide to performing polygenic risk score analyses. *Nature protocols*, 15(9):2759–2772.

Chourpiliadis, C., Zeng, Y., Lovik, A., Wei, D., Valdimarsdóttir, U., Song, H., Hammar, N., and Fang, F. (2024). Metabolic profile and long-term risk of depression, anxiety, and stress-related disorders. *JAMA network open*, 7(4):E244525.

Davies, R. B. (1987). Hypothesis testing when a nuisance parameter is present only under the alternative. *Biometrika*, 74(1):33–43.

Dudbridge, F. (2013). Power and predictive accuracy of polygenic risk scores. *PLoS genetics*, 9(3):e1003348–e1003348.

Gamazon, E. R., Wheeler, H. E., Shah, K. P., Mozaffari, S. V., Aquino-Michaels, K., Carroll, R. J., Eyler, A. E., Denny, J. C., Nicolae, D. L., Cox, N. J., Im, H. K., Consortium, G., and Consortium, G. (2015). A gene-based association method for mapping traits using reference transcriptome data. *Nature genetics*, 47(9):1091–1098.

Gusev, A., Ko, A., Shi, H., Bhatia, G., Chung, W., Penninx, B. W. J. H., Jansen, R., de Geus, E. J. C., Boomsma, D. I., Wright, F. A., Sullivan, P. F., Nikkola, E., Alvarez,

M., Civelek, M., Lusis, A. J., Lehtimäki, T., Raitoharju, E., Kähönen, M., Seppälä, I., Raitakari, O. T., Kuusisto, J., Laakso, M., Price, A. L., Pajukanta, P., and Pasaniuc, B. (2016). Integrative approaches for large-scale transcriptome-wide association studies. *Nature genetics*, 48(3):245–252.

Janssens, A. C. J. W. (2019). Validity of polygenic risk scores: are we measuring what we think we are? *Human molecular genetics*, 28(R2):R143–R150.

Kim, Y. (2025). Cauchygm: Cauchy graphical models in r. R package. Available at: `https://rdrr.io/github/ykim03517/CauchyGM/`.

Lee, S. S., Zhangchen Zhao, w. c. f. L. M., and Wu, M. (2023). *SKAT: SNP-Set (Sequence) Kernel Association Test*. R package version 2.2.5.

Machiela, M. J., Chen, C.-Y., Chen, C., Chanock, S. J., Hunter, D. J., and Kraft, P. (2011). Evaluation of polygenic risk scores for predicting breast and prostate cancer risk. *Genetic epidemiology*, 35(6):506–514.

Mai, J., Lu, M., Gao, Q., Zeng, J., and Xiao, J. (2023). Transcriptome-wide association studies: recent advances in methods, applications and available databases. *Communications biology*, 6(1):899–899.

Meier, S., Strohmaier, J., Breuer, R., Mattheisen, M., Degenhardt, F., Mühleisen, T. W., Schulze, T. G., Nöthen, M. M., Cichon, S., Rietschel, M., and Wüst, S. (2013). Neuregulin 3 is associated with attention deficits in schizophrenia and bipolar disorder. *The international journal of neuropsychopharmacology*, 16(3):549–556.

Muggeo, V. M. R. (2008). Segmented: An r package to fit regression models with broken-line relationships. *R News*, 8:20–25. https://journal.r-project.org/articles/RN-2008-004/.

Paterson, C., Wang, Y., Hyde, T. M., Weinberger, D. R., Kleinman, J. E., and Law, A. J.

(2017). Temporal, diagnostic, and tissue-specific regulation of nrg3 isoform expression in human brain development and affective disorders. *The American journal of psychiatry*, 174(3):256–265.

Simonson, M. A., Wills, A. G., Keller, M. C., and McQueen, M. B. (2011). Recent methods for polygenic analysis of genome-wide data implicate an important effect of common variants on cardiovascular disease risk. *BMC genetics*, 12(1):146–146.

Sollis, E., Mosaku, A., Abid, A., Buniello, A., Cerezo, M., Gil, L., Groza, T., Güneş, O., Hall, P., Hayhurst, J., Ibrahim, A., Ji, Y., John, S., Lewis, E., Macarthur, J. A. L., Mcmahon, A., Osumi-Sutherland, D., Panoutsopoulou, K., Pendlington, Z., Ramachandran, S., Stefancsik, R., Stewart, J., Whetzel, P., Wilson, R., Hindorff, L., Cunningham, F., Lambert, S. A., Inouye, M., Parkinson, H., and Harris, L. W. (2023). The nhgri-ebi gwas catalog: knowledgebase and deposition resource. *Nucleic acids research*, 51(1 D):D977–D985.

Xu, Z., Wu, C., Wei, P., and Pan, W. (2017). A powerful framework for integrating eqtl and gwas summary data. *Genetics (Austin)*, 207(3):893–902.

Yue Hu, Menglu Che, H. Z. (2025). Sex-specific association between polymorphisms in estrogen receptor alpha gene (esr1) and depression: A genome-wide association study of all of us and uk biobank data. *Genetic Epidemiology*. To appear.

Zhao, Z., Gruenloh, T., Yan, M., Wu, Y., Sun, Z., Miao, J., Wu, Y., Song, J., and Lu, Q. (2024). Optimizing and benchmarking polygenic risk scores with gwas summary statistics. *Genome Biology*, 25(1):260–28.
